# Fetal sex modulates placental microRNA expression, potential microRNA-mRNA interactions, and levels of amino acid transporter expression and substrates: INFAT study subpopulation analysis of n-3 LCPUFA intervention during pregnancy and associations with offspring body composition

**DOI:** 10.1186/s12860-021-00345-x

**Published:** 2021-03-03

**Authors:** Eva-Maria Sedlmeier, Dorothy M. Meyer, Lynne Stecher, Manuela Sailer, Hannelore Daniel, Hans Hauner, Bernhard L. Bader

**Affiliations:** 1 ZIEL-PhD Graduate School ‘Epigenetics, Imprinting and Nutrition’, ZIEL-Institute for Food and Health, School of Life Sciences Weihenstephan, Technical University of Munich, Gregor-Mendel-Straße 2, 85354 Freising, Germany; 2 Else Kröner-Fresenius-Center for Nutritional Medicine, School of Life Sciences Weihenstephan, Technical University of Munich, Gregor-Mendel-Straße 2, 85354 Freising, Germany; 3 Institute of Nutritional Medicine, School of Medicine, Technical University of Munich, Georg-Brauchle-Ring 62, 80992 Munich, Germany; 4 Molecular Nutrition Unit, ZIEL-Institute for Food and Health, School of Life Sciences Weihenstephan, Technical University of Munich, Gregor-Mendel-Straße 2, 85354 Freising, Germany; 5 Clinical Nutritional Medicine Unit, ZIEL-Institute for Food and Health, School of Life Sciences Weihenstephan, Technical University of Munich, Gregor-Mendel-Straße 2, 85354 Freising, Germany

**Keywords:** Adipose tissue, Amino acid transport, Fetal programming, MicroRNA, N-3 long-chain polyunsaturated fatty acids, Placenta, Sexual dimorphism

## Abstract

**Background:**

Previously, we revealed sexually dimorphic mRNA expression and responsiveness to maternal dietary supplementation with n-3 long-chain polyunsaturated fatty acids (LCPUFA) in placentas from a defined INFAT study subpopulation. Here, we extended these analyses and explored the respective placental microRNA expression, putative microRNA-mRNA interactions, and downstream target processes as well as their associations with INFAT offspring body composition.

**Results:**

We performed explorative placental microRNA profiling, predicted microRNA-mRNA interactions by bioinformatics, validated placental target microRNAs and their putative targets by RT-qPCR and western blotting, and measured amino acid levels in maternal and offspring cord blood plasma and placenta. microRNA, mRNA, protein, and amino acid levels were associated with each other and with offspring body composition from birth to 5 years of age. Forty-six differentially regulated microRNAs were found. Validations identified differential expression for *microRNA-99a* (*miR-99a*) and its predicted target genes *mTOR*, *SLC7A5*, encoding L-type amino acid transporter 1 (LAT1), and *SLC6A6*, encoding taurine transporter (TauT), and their prevailing significant sexually dimorphic regulation. Target mRNA levels were mostly higher in placentas from control male than from female offspring, whereas respective n-3 LCPUFA responsive target upregulation was predominantly found in female placentas, explaining the rather balanced expression levels between the sexes present only in the intervention group. LAT1 and TauT substrates tryptophan and taurine, respectively, were significantly altered in both maternal plasma at 32 weeks’ gestation and cord plasma following intervention, but not in the placenta. Several significant associations were observed for *miR-99a, mTOR* mRNA, *SLC7A5* mRNA, and taurine and tryptophan in maternal and cord plasma with offspring body composition at birth, 1 year, 3 and 5 years of age.

**Conclusions:**

Our data suggest that the analyzed targets may be part of a sexually dimorphic molecular regulatory network in the placenta, possibly modulating gene expression per se and/or counteracting n-3 LCPUFA responsive changes, and thereby stabilizing respective placental and fetal amino acid levels. Our data propose placental *miR-99*, *SLC7A5* mRNA, and taurine and tryptophan levels in maternal and fetal plasma as potentially predictive biomarkers for offspring body composition.

**Supplementary Information:**

The online version contains supplementary material available at 10.1186/s12860-021-00345-x.

## Background

The obesity epidemic, especially in children, is an enormous public health and economic burden [1, 2]. The concept of fetal programming or developmental origins of health and disease has drawn the attention to pregnancy and lactation as critical windows for early prevention of obesity [3–5]. Based on in vitro studies and animal and human studies adipose tissue development can be altered by a decreased nutritional n-6/n-3 LCPUFA ratio during pregnancy and lactation [6] suggesting a novel primary prevention strategy for childhood obesity in humans. In this context, we previously conducted the randomized controlled intervention study INFAT (impact of nutritional fatty acids during pregnancy and lactation on early human adipose tissue development) to investigate whether a reduced maternal n-6/n-3 LCPUFA ratio during pregnancy is a primary prevention strategy against offspring obesity [7]. Many opposing effects of n-6 and n-3 fatty acids are mediated through altered gene expression [8]. Mechanistically, PUFAs can affect gene expression through membrane composition changes, raising second messenger concentrations and eicosanoid production [9]. Furthermore, PUFAs can act via their various metabolites in conjunction with G-protein coupled receptors, nuclear receptors (e.g. PPARα-γ) and transcription factors (e.g. SREBP-1c or NFκB) on gene expression [8, 9]. Our previous mRNA transcriptome analyses of human placentas from a defined INFAT study subpopulation showed sexually dimorphic gene expression and responsiveness to maternal dietary n-3 LCPUFA supplementation [10]. It has also been demonstrated that PPARγ agonists, such as n-3 LCPUFAs and metabolites including 15d-PGJ2, can stimulate the mechanistic target of rapamycin (mTOR) serine/threonine kinase and nutrient transporter expression in placental trophoblasts, indicating their involvement in controlling fetal growth [11]. Signalings through mTOR complex 1 (mTORC1), which includes the mTOR protein, are key pathways for nutrient availability sensing and growth factor signaling as well as regulating cell growth and metabolism by modulating gene expression and protein translation [12].

The fetus depends primarily on placenta-mediated nutrients, highlighting the essential role of the placenta in fetal growth and development [13–15]. Therefore, changes in placental nutrient transport and hormones influence fetal metabolism, cell differentiation, and organ development including adipogenesis [16, 17]. Interestingly, several in vitro studies have shown that PUFA treatment also resulted in expression changes of microRNAs [18–20], mediating post-transcriptional regulation of gene expression [21–23]. In this context, treatment with n-3 LCPUFA docosahexaenoic acid (DHA) and n-6 LCPUFA arachidonic acid (AA) can differentially regulate the microRNA expression pattern [18]. Moreover, DHA, but not saturated fatty acid, can significantly increase the levels of specific microRNAs in a dose-dependent fashion [20]. In addition, it has been reported that microRNAs are involved in adipocyte differentiation and show differences in adipogenesis between lean and obese subjects [24–27]. However, it is unknown whether similarities in microRNA-mRNA interactions and n-3 LCPUFA target genes exist between placental and adipose tissues or in other organs, such as muscle tissue. The importance of microRNA in the placenta is indicated by the high abundance of placenta-specific microRNAs and the necessity of an intact microRNA machinery for proper placental development [28–30].

We recently reported on several analyses of the INFAT population (*n* = 208) that explored adipose tissue development in relation to fetal programming and offspring obesity risk [7, 10, 31–34], including a report on sex-specific placental gene regulation and function in a defined INFAT subpopulation of mother-offspring pairs (*n* = 41) [10]. The present secondary analysis took advantage of the mRNA, microRNA, and protein samples derived from the same placental specimens of the aforementioned INFAT subpopulation [10] and offered a unique opportunity to explore the impact of maternal n-3 LCPUFA supplementation on placental microRNA, mRNA, and protein expression, and potential microRNA-mRNA interactions and downstream targets.

Here, we examined the transcriptional regulation of placental nutrient transport, including sex-specific expression, and their associations with offspring anthropometric parameters, including adipose tissue and lean mass, from birth up to 5 years of life.

**Fig. 1:**
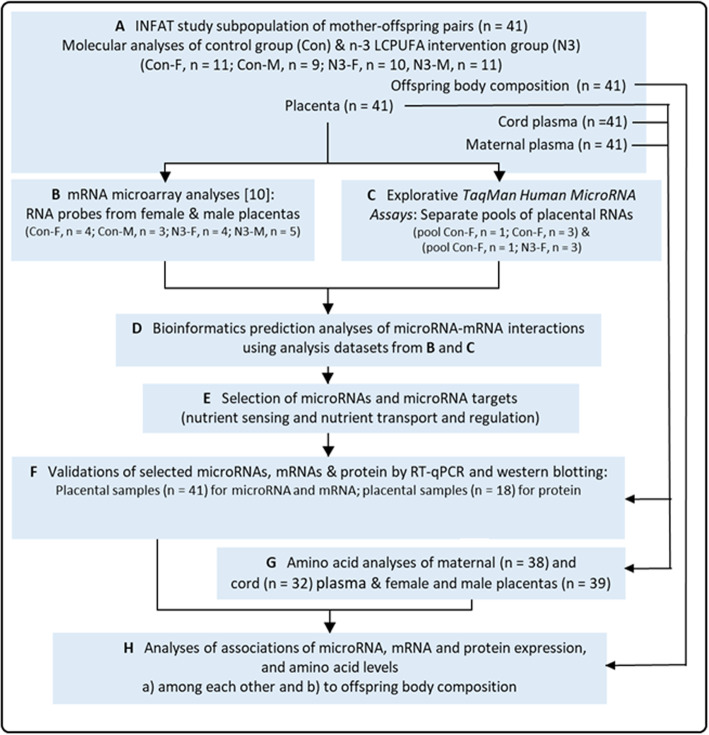
Flowchart of analyses. **a**, **b** Maternal and umbilical cord blood samples, placentas and offspring anthropometric parameters from mother-offspring pairs of the subpopulation control (*n* = 20) and n-3 LCPUFA intervention group (*n* = 21) of the INFAT cohort, as well as respective placental mRNA transcriptome datasets from our previous study [10] were used in this study for molecular analyses (microRNA, mRNA, protein, amino acids), bioinformatics and association analyses as depicted (**b - h**)

Based on our previous discovery that n-3 LCPUFA treatment predominantly changed gene expression in female offspring placentas [10], we began the present INFAT study subpopulation analyses (see analyses flowchart in Fig. 1) with an explorative placental microRNA profiling *by 384-well TaqMan low density human microRNAs assays* using group-specific pools (intervention and control) representing microRNA samples from three female offspring placentas in each group. It is important to note that we have specifically chosen samples of total RNA with small RNAs that were isolated from female placenta specimens included in our previous mRNA transcriptome analyses [10]. This ensured that the present microRNA profiling data and the previous mRNA transcriptome data were compatible for the subsequent microRNA-mRNA interaction analyses. By combining the datasets from placental microRNA profiling and mRNA transcriptome analyses [10], we analyzed microRNAs, which were differentially expressed between the intervention and control group, for their putative binding to n-3 LCPUFA-regulated mRNAs. Using a targeted approach, a selection of identified microRNAs and their predicted microRNA target genes related to nutrient sensing and amino acid transport and regulation were further stepwise validated using female and male placentas (*n* = 41) from the INFAT subpopulation by reverse transcription real-time quantitative PCR (RT-qPCR) and western blot experiments. MicroRNAs, identified by microRNA profiling, were selected for subsequent validation analyses of their respective target mRNAs and proteins, if they showed significant group- and sex-specific expression and n-3 LCPUFA-responsiveness by RT-qPCR validations. Correlation analyses were then performed to explore relationships among validated microRNA and target mRNA and protein levels, as well as whether sex-steroid hormones are potentially involved in the observed sex-specific placental gene expression. To analyze a potential biological read-out of the observed expression regulation for amino acid transporters, we first examined their substrate amino acid levels in placental specimens as well as maternal and cord plasma. Finally, association analyses were performed to explore the potential effects of the identified placental gene and protein expression levels and amino acid concentrations both among each other and with offspring anthropometric parameters at birth and 1 year, 3 and 5 years of age.

## Results

### Maternal and offspring clinical characteristics of the INFAT study subpopulation and specifics on placental specimens and respective mRNA, microRNA, and protein samples

The mother-offspring pairs (*n* = 41) from the defined INFAT study subpopulation described in our previous report on placental mRNA expression [10] are identical to those in the present study. For convenience, the clinical characteristics for mothers and their offspring of the n-3 LCPUFA intervention group (N3, *n* = 21; female *n* = 10, male = 11) and control group (Con, *n* = 20; female *n* = 11, male = 9) as already described in our previous report [10] are shown in Additional file 1: Table S1 in the present report. The characteristics of the INFAT study subpopulation largely resemble the data of the whole INFAT study population (*n* = 208) which demonstrated that the n-3 LCPUFA intervention effectively reduced the n-6/n-3 LCPUFA ratio as expected [12, 34]. In addition, no adverse effects for mothers or offspring were observed in the study population. For our analyses, mRNA, microRNA, protein extracts, and amino acid data were derived from the same placenta-specimen starting material (chorionic villous portion) for each INFAT subgroup participant. Moreover, to match mRNA and microRNA samples as closely as possible, their extraction and preparation were done from the same *TRIzol*-based starting placenta homogenate. In addition to these samples, respective plasma samples from maternal and cord blood of the mother-offspring pairs (*n* = 41) were used for analyses.

### Explorative microRNA profiling of placentas

Our previous mRNA transcriptome analyses showed n-3 LCPUFA responsive mRNA expression changes predominantly in placentas from female offspring [10]. Consequently, for explorative placental microRNA profiling (*384-well TaqMan low density human microRNA assays*) microRNAs from female offspring placentas of the INFAT subpopulation intervention (*n* = 3) and control (*n* = 3) groups were used, which were already analyzed for mRNA expression in our previous transcriptome analyses [10], and thereby qualifying for the analyses of microRNA-mRNA interactions using respective datasets. Equal amounts of total RNA with small RNA from three female placentas per group were pooled for cDNA synthesis and amplification. Group-specific pools (*n* = 1 per group) then were analyzed in parallel by real time quantitative PCR arrays (*TaqMan low-density human microRNA assays*). This experimental design was taken to reduce the cost and complexity of analysis of our explorative microRNA profiling but should allow for the identification of the most common differentially expressed microRNAs following n-3 LCPUA supplementation. This experimental design cannot provide complete group representation and therefore represents a known initial limitation of our study.

**Fig. 2:**
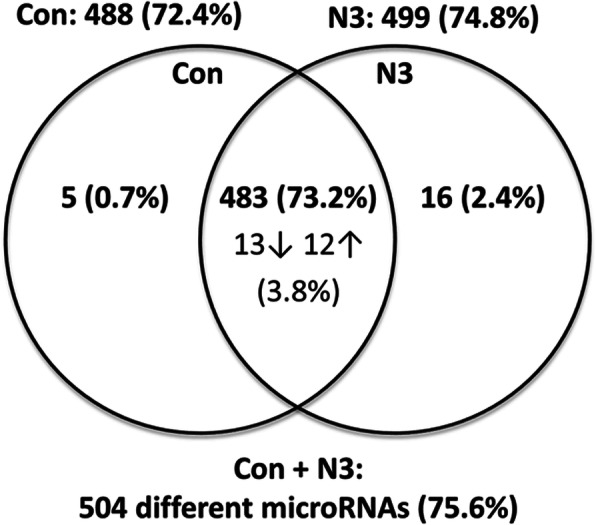
Venn diagram of placental microRNAs identified by microRNA profiling. Venn diagram showing the numbers of detected and differentially expressed microRNAs identified in pooled probes from female placentas of each group (Con, control group; N3, intervention group) by *qPCR TaqMan low density human microRNA assay*. Each pool (n = 1) representing 3 female placentas from each group was screened for 667 human microRNAs. Venn diagram represents the number of separately detected microRNAs in each group and the number of different microRNAs in both groups together (numbers outside of the circles). Total number of different microRNAs are separated in (1) microRNAs detected in one group but not in the other group (numbers exclusively inside the group-specific circle but outside of the intersection and (2) microRNAs common in both groups (numbers in the circle intersection). Within the group of microRNAs common in both groups, the numbers for microRNAs showing differential expression levels between the groups are depicted with upwards and downwards arrow for upregulation and downregulation of microRNAs of the intervention versus control group, respectively. Percent values for respective numbers of detected microRNAs related to 667 microRNAs (total number of screened microRNAs) for each group are shown in parentheses

Overall, from 667 unique human microRNAs screened by qPCR-based *TaqMan low density human microRNA assays*, the presence of 488 microRNAs (72.4%) and 499 microRNAs (74.8%) were detected for the female placenta pools in the control and intervention groups, respectively (Fig. 2). In total, 504 (75.6%) different microRNAs were detected for the pools of both groups and 483 microRNAs (73.2%) were common in the control and intervention groups. Furthermore, from the 504 detected microRNAs, 46 microRNAs, representing 6.9% of all screened microRNAs, showed differential amplification by qPCR (Fig. 2; microRNAs and Cq values are listed in Additional file 2: Table S2). Thereof 21 microRNAs were unique for each group and were classified as expressed (+) or not expressed (−) in the respective female offspring group (Additional file 2 Table S2; N3+: 16 microRNAs; Con+: 5 microRNAs). Regarding differential regulation of microRNAs, from the 483 microRNAs common to both groups, 25 were differentially expressed (12 upregulated microRNAs, 13 downregulated microRNAs), representing 3.8% of all screened microRNAs, as shown in Fig. 2. In addition, from the 504 detected microRNAs 43 belonging to the *chromosome 19 microRNA cluster* (*C19MC*) were identified (Additional file 2: Table S3). *C19MC* microRNAs are exclusively expressed in the placenta and undifferentiated cells [35] and are the most abundant microRNAs in human term primary trophoblast cells [35]. The detected *C19MC* microRNAs showed Cq-values between 11.8 and 30.3 (Additional file 2: Table S3) and all 43 *C19MC* microRNAs were present among the 483 microRNAs common in both groups. Two out of 43 *C19MC* microRNAs, *miR-517b* and *miR-522,* are differentially regulated between the groups and present among the 25 differentially regulated microRNAs mentioned above (Fig. 2, Additional file 2: Table S2 and S3).

### Selection of microRNAs and *miR-99a* targets for validation

Assuming that female placentas express female-specific microRNAs and mRNAs in addition to the microRNAs and mRNAs that are present in placentas of both sexes, mRNAs which show respective n-3 LCPUFA responsive regulation in our previous mRNA microarray datasets from the subgroups of INFAT female and male placentas [10] were chosen to find binding sites for regulated microRNAs on respective differential regulated placental mRNAs in both sexes. *DIANA mirExTra* application was used to examine which of the 46 differentially expressed microRNAs, identified in microRNA pools of female placentas by microRNA profiling (Additional file 2: Table S2), possess respective seed-sites (nucleotide 2–8 of microRNA) in 459 significantly differentially regulated mRNAs upon n-3 LCPUFA intervention identified by our previous mRNA microarray analyses [10]. This set of mRNAs was chosen because it represents all significantly regulated genes from three microarray datasets examined for the effect of n-3 LCPUFA intervention (intervention versus control group analyzed separately in female and male placentas and adjusted for offspring sex; see n-3 LCPUFA intervention datasets in [10]) and thereby covering mRNAs expressed not only in female, but also in male placentas. In the present study, we found 23 microRNAs with significantly higher *DIANA microT scores* (*p* < 0.05) reflecting that their seed-sites are overrepresented in these differentially expressed mRNAs upon intervention (Table 1). Thereof, based on bioinformatics and published literature, microRNAs *miR-30d, miR-99a, miR-100*, and *miR-320* were selected for further analyses due to their seed-sites in potential mRNA targets (Table 2) and putative involvement in nutrient sensing and transport, e.g. amino acid and glucose transport as follows. In the present study, in the SLC6A6 gene, encoding the taurine transporter TauT [11, 12], a *miR-30d* seed-site was identified by *DIANA mirExTra* (Table 2). *miR-320* can target the gene *PIK3R1* encoding phosphatidylinositol 3-kinase p85, that is involved in the regulation of the glucose transporter GLUT4 in insulin-resistant 3T3-L1 adipocytes [36]. *MiR-99a* and *miR-100* were both selected since both can target *mTOR* expression [37]. mTOR is a Ser/Thr kinase found in two protein complexes named the mTOR complex 1 (mTORC1) and mTOR complex 2 (mTORC2) and mTORC1 plays an important role in not only nutrient sensing, but also in cell cycle progression, cell growth, lipid and protein synthesis, autophagy, and energy metabolism [16, 17]. *mTOR* represents a functionally validated *miR-99a* target in several cancer cell lines and primary cells, but the interaction between *miR-99a* and *mTOR* expression or mTORC1 activity was not investigated in placental tissue or cell lines thus far [37–39]. Importantly, mTORC1 can regulate the placental amino acid transporter LAT1 *and* TauT [40]. To obtain additional information on putative *miR-99a* seed-site sequences in non-coding and coding nucleotide sequences for *mTOR* and the genes *SLC7A5* and *SLC6A6*, encoding the amino acid transporter LAT1 and TAUT, respectively, we applied additional bioinformatics data analyses, including various prediction algorithms (*DIANA-microT-CDS*, *DIANA-microT*, *microRNA.org*, *miRDB*, *TARGETMINER*, *TARGETSCAN-vert*, or *PICTAR-vert*), searched databases storing information for experimentally identified microRNA targets (*miRecords*, *miRSel*, *miRWalk*, *StarBase*) [41], and conducted sequence alignments [23]. Coding sequences of the target genes were also included in our sequence alignments, since a new class of microRNA-recognition elements (MRE) can function exclusively in the target protein coding sequences mediating translational control [42, 43]. Table 2 lists predicted 5′ seed-sites with a complete match or matches with 1 or 2 mismatches in the respective target genes and the putative MRE-sites. In addition to the reported *miR-99a* functional seed-site in the *mTOR* 3′-UTR, three further matches in the *mTOR* coding region were found. A *miR-99a* seed-site with two mismatches for *SLC6A6*, predicted by the microRNA target prediction algorithm *rna22* [44], was found in the 3′-UTR of *SLC6A6*. An *miR-99a* seed-site 98 nucleotides 5′-adjacent to the 3′-UTR was determined for *SLC7A5*. In addition, we detected two sequence elements in the coding sequence reflecting 5′ seed-sites with two mismatches and sequence elements with a potential for a moderate 3′-site base-pairing MRE with *miR-99a* in exon 1 of *SLC7A5*. Considering the various predicted *miR-99a* seed-sites in the target genes *mTOR*, *SLC7A5* and *SLC6A6* and their important roles in amino acid transport and its regulation in the placenta [40], they were among the potential candidates for further expression validation, provided that respective selected microRNAs were validated as significantly regulated by subsequent RT-qPCR experiments using samples from female and male placentas of the whole INFAT study subpopulation (*n* = 41).

**Table 1:** *DIANAmiR-ExTra* data: n-3 LCPUFA regulated placental microRNAs with predicted target sites on validated target genes

MicroRNA	Fold change	Median Cq	***P***	Genes over threshold
*hsa-miR-888*	−11.45	34.0	7.29 E-05	33
*hsa-miR-375*	−6.77	25.1	2.12 E-03	4
*hsa-miR-586*	−4.50	32.0	6.45 E-05	1
*hsa-miR-130b*	−2.13	21.5	4.66 E-02	22
***hsa-miR-320***	**−1.40**	**16.9**	**2.16 E-03**	**13**
*hsa-miR-21*	−1.36	15.2	9.12 E-06	11
*hsa-miR-522*	−1.32	15.6	8.72 E-04	10
***hsa-miR-30d***	**1.29**	**16.6**	**5.38 E-03**	**47**
*hsa-miR-451*	1.30	15.2	2.61 E-02	0
*hsa-miR-495*	1.35	17.8	1.22 E-12	63
*hsa-miR-517b*	1.41	17.1	5.16 E-04	43
*hsa-miR-139-5p*	1.51	18.8	7.22 E-03	13
***hsa-miR-99a***	**1.53**	**17.5**	**1.00 E-02**	**16**
***hsa-miR-100***	**1.55**	**15.5**	**4.40 E-03**	**15**
*hsa-miR-668*	3.14	26.0	1.73 E-04	6
*hsa-miR-641*	3.39	30.9	1.68 E-03	8
*hsa-miR-302b*	21.22	37.4	3.32 E-03	18
*hsa-miR-367*	Con (+) / N3 (−)	34.0†	7.80 E-05	23
*hsa-miR-649*	Con (+) / N3 (−)	33.6†	8.78 E-03	2
*hsa-miR-302a*	Con (+) / N3 (−)	36.4†	1.47 E-02	21
*hsa-miR-569*	Con (−) / N3 (+)	35.6†	1.57 E-03	2
*hsa-miR-630*	Con (−) / N3 (+)	32.5†	2.47 E-02	27
*hsa-miR-155*	Con (−) / N3 (+)	32.1†	4.65 E-02	10

Using *DIANAmiR-ExTra* 23 n-3 LCPUFA-regulated placental microRNAs with potential target sites on validated differentially expressed placental target genes following n-3 LCPUFA supplementation (N3) were found. Differentially expressed genes were identified by our previous mRNA microarray analyses (n-3 LCPUFA data sets) [10]. n-3 LCPUFA-regulated placental microRNAs with respective median Cq-values and expression fold changes compared to controls (Con) are listed. Number of significantly n-3 LCPUFA regulated genes with predicted potential binding-sites over threshold for the respective microRNAs by *DIANAmiR-ExTra* are depicted. *P*-value calculated by one-sided Wilcoxon rank sum test; microRNAs selected for further validation are marked in bold. Cq, quantification cycle; microRNA expressed in one group (+) but not in the other group (−); †, median Cq not calculated

**Table 2:**
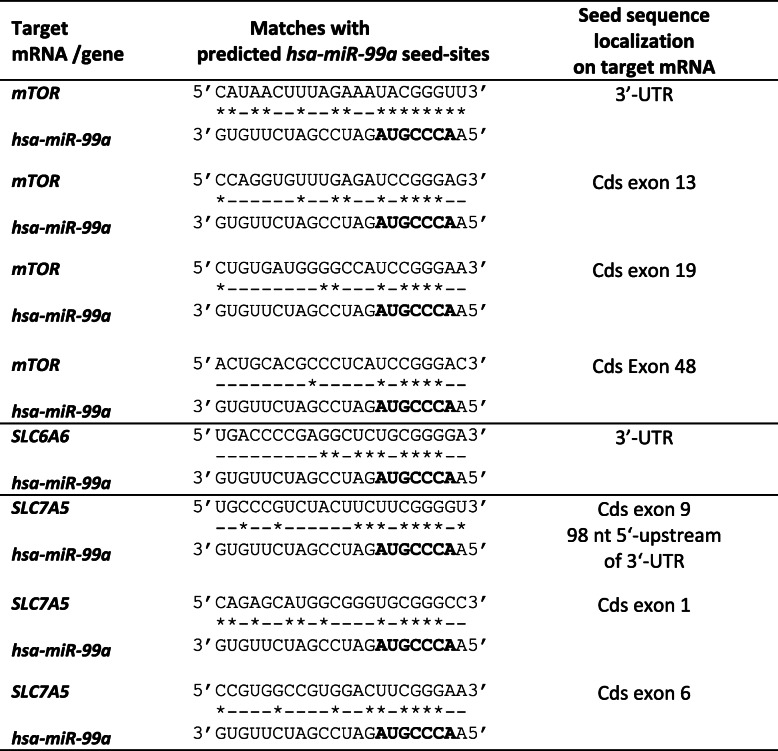
Predicted sequences on target mRNAs complementary to seed-sites of *hsa-miR-99a*

Sequence alignments of human microRNA *hsa-miR-99a* with putative mRNA target sequences are shown. Seed sequence in *hsa-miR-99a* is shown in bold and asterisks mark complementary nucleotide matches with target mRNAs. Cds, coding sequence; 3′-UTR, 3′ untranslated region

### Expression validation reveals sex-specific placental expression and responsiveness to n-3 LCPUFA

**Fig. 3:**
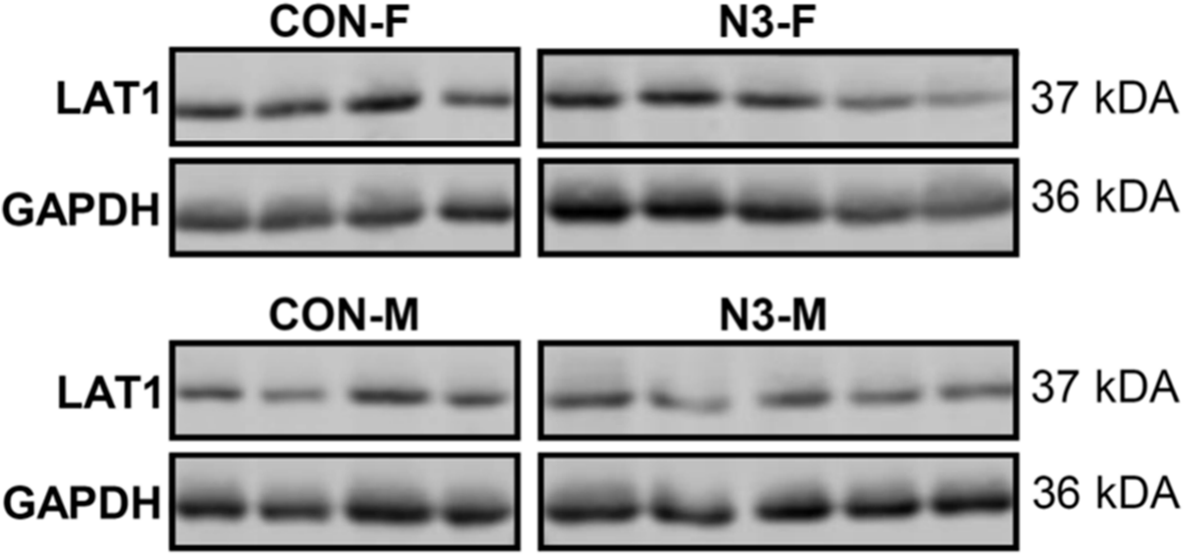
Placental LAT1 protein expression. Western blot analysis of placental LAT1 and GAPDH protein levels from female (Con-F) and male (Con-M) offspring of the control group and female (N3-F) and male (N3-M) offspring of the n-3 LCPUFA intervention group are shown (for total blots see Additional file 3: Figure S3). GAPDH was used for protein normalization. Relative optical densities were calculated with the mean for the respective groups and relative expression levels and effects for group, sex, and interaction of group and sex are shown in Table 3

For all further validation experiments, respective microRNA, mRNA, and protein samples derived from female (*n* = 21) and male (*n* = 20) offspring of control and n-3 LCPUFA subgroups were used. *miR-30d, miR-100*, *miR-99a,* and *miR-*320 were first validated for differential and sex-specific expression by RT-qPCR. Treatment effects between n-3 LCPUFA intervention and control groups were analyzed, independent and dependent of the term “sex”. Furthermore, the applied group interaction term examined whether the effect of the intervention differed according to sex or whether expression differences exist between the sexes without the intervention, using the data from the control group. As summarized in Table 3 (see respective scatter plots in Additional file 3: Figure S1), significant effects were only found for *miR-99a*, but not for *miR-30d*, *miR-100*, and *miR-320*. *miR99-a* levels were significantly higher in the intervention than the control group (1.49-fold, *p* = 0.001). Within the control group, *miR-99a* levels in male placentas were significantly higher than for female placentas (1.42-fold, *p* = 0.039), whereas only the female placentas in the intervention group showed significantly elevated levels (1.86-fold, *p* < 0.001). Next, *miR-99a* was selected for further validation and we focused our microRNA target validation on potential *miR-99* targets *mTOR*, *SLC7A5* and *SLC6A* 3 (Table 3, see respective scatter plots in Additional file 3: Figure S1 and S2)*,* and LAT1 protein (Table 3, Fig. 3, Additional file 3: Figure S3). We hypothesized that upregulated *miR-99a* mediates downregulation of its mRNA targets and /or decreases respective proteins. Target mRNA expression was validated for all targets by RT-qPCR, whereas the protein expression of amino acid transporter LAT1, encoded by *SLC7A5*, was validated exemplarily by western blotting for the effect of microRNA on respective target protein expression. Our expression analyses and exploratory pairwise comparisons of the regression analyses revealed: sex-specific expression differences in the control group and in all placentas from both groups (without the factor treatment), n-3 LCPUFA treatment effects between control and intervention group, and sex-dependent effects following n-3 LCPUFA treatment (Table 3). *SLC7A5* mRNA levels were significantly higher in male than female placentas in the control group (1.83-fold, *p* = 0.001) and *mTOR* mRNA levels tended to be significantly elevated in male compared to female placentas (1.19-fold, *p* = 0.62), whereas LAT1 protein was significantly lower in male than female placentas (1.41-fold, *p* = 0.010). *SLC6A6* mRNA was not differentially expressed between sexes. Exploring the sex-specific differences in both groups together without intervention term, *SLC7A5* mRNA was significantly elevated in male versus female placentas (1.34-fold, *p* = 0.019), whereas LAT1 protein was significantly decreased in male versus female placentas (1.35-fold, *p* = 0.004). Comparing the intervention to the control group, *mTOR* mRNA levels were significantly higher (1.22-fold, *p* = 0.008) in the presence of an n-3 LCPUFA intervention, whereby *SLC6A6* mRNA (1.89-fold, *p* < 0.001) and LAT1 (1.35-fold, p = 0.010) were significantly lower. In addition, *SLC7A5* mRNA levels tended to be significantly increased (1.22-fold, *p* = 0.052). With regard to sex-specific n-3 LCPUFA responsiveness of target molecules related to control female levels, predominantly significant changes were observed in female but not in male intervention group placentas. Additionally, *mTOR* mRNA (1.36-fold, *p* = 0.003) and *SLC7A5* mRNA (1.65-fold, *p* = 0.002) were significantly upregulated and LAT1 was significantly downregulated (1.35-fold, *p* = 0.014) in female intervention group placentas, leading to rather balanced expression levels between the sexes in the intervention group in contrast to sex-specific expression differences found in the control group. However, significantly decreased expression of *SLC6A6* mRNA in both male (2.0-fold, *p* < 0.010) and female (1.75-fold, p < 0.010) placentas were observed in the intervention group.

**Table 3:** Data on validated expression of microRNAs, mRNAs, and protein

	Control			Intervention			Control						Intervention						***P***-values regression						
	(Con-F + Con-M)			(N3-F + N3-M)			Con-F			Con-M			N3-F			N3-M			Treatment	Sex	Interaction	Con-M vs Con-F	N3-M vs N3-F	N3-F vs Con-F	N3-M vs Con-M
	N	MRE		N	MRE		N	MRE		N	MRE		N	MRE		N	MRE								
		%	± SD		%	± SD		%	± SD		%	± SD		%	± SD		%	± SD							
**microRNA**																									
*miR-30d*	16	100 ± 30		19	97 ± 33		9	100 ± 35		7	97 ± 29		10	94 ± 38		9	98 ± 31		0.620	0.756	0.652	0.899	0.596	0.506	0.997
*miR-320*	18	100 ± 64		19	108 ± 61		10	100 ± 70		8	92 ± 59		10	111 ± 68		9	95 ± 57		0.415	0.515	0.650	0.903	0.438	0.369	0.831
*miR-100*	19	100 ± 34		18	107 ± 40		11	100 ± 33		8	108 ±37		9	115 ± 41		9	107 ± 42		0.558	0.917	0.389	0.594	0.492	0.313	0.812
*miR-99a*	19	**100 ± 51**		19	**149 ± 70**		11	**100 ± 41**		8	**142 ± 63**		10	**186 ± 70**		9	164 ± 79		**0.001**	0.511	**0.026**	**0.039**	0.266	**< 0.001**	0.452
**mRNA**																									
*mTOR*	20	**100 ± 22**		21	**122 ± 34**		11	**100 ± 13**		9	**119 ± 21**		10	**136 ± 38**		11	129 ± 28		**0.008**	0.330	0.098	0.062	0.649	**0.003**	0.441
*SLC7A5*	20	**100 ± 71**		21	**122 ± 70**		11	**100 ± 60**		9	**183 ± 104**		10	**165 ± 80**		11	170 ± 90		0.052	**0.019**	**0.012**	**0.001**	0.955	**0.002**	0.704
*SLC6A6*	20	**100 ± 60**		20	**53 ± 25**		11	**100 ± 40**		9	**109 ± 67**		9	**57 ± 22**		11	**54 ± 18**		**< 0.001**	0.945	0.807	0.825	0.900	**< 0.001**	**< 0.001**
**Protein**																									
LAT1	8	**100 ± 25**		10	**77 ± 17**		4	**100 ± 25**		4	**71 ± 17**		5	**74 ± 21**		5	58 ± 16		**0.010**	**0.004**	0.341	**0.010**	0.085	**0.014**	0.183

Validation data of selected placental microRNAs from microRNA profiling and of target mRNAs by qPCR (see respective scatter plots Additional file 3: Figure S1 and S2) and of placental LAT1 (normalized to GAPDH protein, see Fig. 3 and Additional file 3: Figure S3) by western blot are shown. The expression level of the female control offspring (Con-F) samples was assigned an arbitrary value of 100% and all other analyzed groups (Con-M, male offspring of the control group; N3-F and N3-M, female and male offspring of the intervention group, respectively) were calculated relative to Con-F. Data are presented as mean relative expression (MRE) in % + standard deviation (SD). The *P*-values for *Treatment* and *Sex* come from linear regression models fit to the expression data without a *Sex-Group Interaction* term. The *P*-values for the *Interaction* and pairwise group comparisons (Con-M vs Con-F, N3-M vs N3-F, N3-F vs Con-F, N3-M vs CM) are from regression models including a *Sex-Group Interaction* term. *P*-values *p* < 0.05 were considered as significant and are shown in bold

### Associations of expression levels for *miR-99a* and their potential *miR-99a* targets among each other

To assess whether *miR-99a* and its potential mRNA targets are part of a regulated expression network, we estimated their associations among each other. In general, inverse associations exist between microRNAs and their target mRNAs upon microRNA-primed target mRNA degradation [21–23]. However, microRNAs can also mediate translational control without target mRNA degradation and therefore inverse associations are not found [42–44]. In the present study, we found mainly significant moderate associations. *MiR-99a* was significantly inversely associated with *SLC6A6* mRNA (*p* = 0.009) only, but significantly positively associated with *mTOR* (*p* = 0.033) and *SLC7A5* (*p* = 0.006) mRNA levels (Table 4). Furthermore, *mTOR* mRNA was significantly positively associated with *SLC7A5* mRNA (*p* = 0.003), but not with LAT1 protein (*p* = 0.101), and significantly negatively associated with *SLC6A6* mRNA (*p* = 0.033). For *SLC6A6* mRNA significant inverse associations with *SLC7A5* mRNA (*p* = 0.049) and a positive association with LAT1 (*p* = 0.010) were observed.

**Table 4:** Correlations among regulated levels of placental microRNA, mRNA, and protein expression

		***miR-99a***	***mTOR***	***SLC7A5***	LAT1
***mTOR***	Rs	***0.35***			
	*P*	**0.033**			
	N	38			
***SLC7A5***	Rs	**0.43**	**0.46**		
	*P*	**0.006**	**0.003**		
	N	38	41		
**LAT1**	Rs	−0.39	−0.40	−0.09	
	*P*	0.131	0.101	0.735	
	N	16	18	18	
***SLC6A6***	Rs	**−0.42**	**− 0.34**	**− 0.11**	**0.59**
	*P*	**0.009**	**0.033**	**0.049**	**0.010**
	N	37	40	40	18

The association analyses were conducted independent of n-3 LCPUFA intervention status. ΔCq-values for mRNA and microRNA expression of respective genes were used; optical density values for LAT1 protein expression normalized to respective GAPDH protein from western blot experiments were used. Positive or negative signs indicate a positive or inverse association, respectively. Rs-values with *P*-values *p* < 0.05 are shown in bold. N, number of analyzed placentas; *P*, *p*-value for the respective correlation; Rs, Spearman-rho correlation coefficient

### Associations of levels steroid hormone levels in placenta and cord plasma with target expression levels

Steroid hormones are major determinants of sexual dimorphism [45, 46]. We therefore previously analyzed steroid hormones in the INFAT study subpopulation as potential candidates to explain underlying mechanisms of sexually dimorphic placental mRNA expression [10]. Respective analysis details, including methods and references, as well as data on all analyzed steroid hormones are described in Sedlmeier et al. [10] and therein see Additional file 1: Table S8. For convenience, data on free estradiol-17ß (E2), testosterone (T), and E2/T ratio are summarized in Additional file 4: Table S4 of this study. As reported, the placental concentrations for E2 and T, and respective E2/T ratio as indirect marker for E2-to-T conversion by aromatase CYP19A1 were not significantly different between the sexes in the control group (Additional file 4: Table S4). Sex-specific effects however were observed in the intervention group. T but not E2 was significantly higher in female than male placentas (*p* = 0.008) and consequently E2/T ratios were significantly lower in female versus male placentas (*p* = 0.002). Comparing female placentas between the intervention and control group, E2/T ratios were significantly increased in the presence of n-3 LCPUFA supplementation. In the present study, we used these previous data for correlation analyses between placental E2, T, including E2/T ratio and *miR-99a*, *mTOR* mRNA, *SLC6A6* mRNA, and *SLC7A5* mRNA (Table 5). No correlations between steroid hormones and placental gene expression levels were found analyzing all placentas together independent of the terms sex and intervention (Table 5, see All offspring). However, moderate significantly positive correlations, that were sex-specific, were observed by analyzing separately all female and male placentas independent of the treatment term (Table 5). E2 significantly correlated with *SCL7A5* mRNA in male placentas (*p* = 0.039; Table 5, see Male offspring). This is interesting, since *SLC7A5* mRNA levels are significantly higher in male than female placentas in the control group per se (*p* = 0.001) and in addition in all male than female placentas (*p* = 0.019) analyzed independent of the treatment term (Table 3). In contrast, E2 or E2/T ratio did not correlate with *SLC7A5* mRNA levels in the other analyzed groups. Further sex-specific but treatment-independent effects were observed (Table 5, see Female offspring and Male offspring). T almost significantly correlated with *miR-99a* (*p* = 0.05) that is consistent with significantly higher *miR-99a* levels in female placentas after n-3 LCPUFA supplementation (*p* < 0.001; Table 3). Moreover, E2/T tended to significantly correlate with *miR-99a* in male placentas (*p* = 0.073). E2/T ratio tended to significantly correlate with *SLC6A6* (*p* = 0.059) in female placentas, consistent with higher levels placental T levels for female versus male offspring (Additional file 4: Table S4) and lower expression of *SLC6A6* mRNA following intervention (Table 3). No correlations between E2, T, and E2/T ratio and *mTOR* mRNA were found.

**Table 5:** Correlations of placental free estradiol-17ß (E2), testosterone (T), and E2/T ratio with levels of target microRNA and mRNA

Group		***mTOR***			***SLC6A6***			***SLC7A5***			***MiR-99a***		
		E2	T	E2/T	E2	T	E2/T	E2	T	E2/T	E2	T	E2/T
**All offspring**	**Rs**	0.124	0.014	0.048	−0.071	−0.081	0.030	0.242	−0.022	0.124	0.107	−0.071	0.092
	**P**	0.441	0.931	0.766	0.665	0.619	0.855	0.127	0.893	0.441	0.523	0.673	0.583
	**N**	41	41	41	40	40	40	41	41	41	38	38	38
**Male** **offspring**	**Rs**	0.251	−0.056	0.197	−0.147	−0.098	−0.161	**0.465**	0.084	0.356	0.061	−0.360	0.446
	**P**	0.286	0.816	0.405	0.535	0.682	0.498	**0.039**	0.724	0.123	0.815	0.155	0.073
	**N**	20	20	20	20	20	20	20	20	20	17	17	17
**Female** **offspring**	**Rs**	0.029	0.183	−0.131	0.093	−0.301	0.429	−0.019	0.147	−0.316	0.027	0.432	−0.343
	**P**	0.902	0.427	0.571	0.696	0.198	0.059	0.933	0.526	0.163	0.909	0.050	0.128
	**N**	21	21	21	20	20	20	21	21	21	21	21	21
**Con:** **All offspring**	**Rs**	0.247	−0.150	0.338	−0.120	−0.053	−0.102	0.235	−0.176	0.116	0.140	−0.267	0.186
	**P**	0.295	0.528	0.145	0.613	0.825	0.668	0.319	0.458	0.627	0.567	0.269	0.446
	**N**	20	20	20	20	20	20	20	20	20	19	19	19
**N3:** **All offspring**	**Rs**	0.073	0.110	−0.026	−0.104	−0.035	−0.038	0.204	−0.051	0.238	0.096	0.011	0.065
	**P**	0.754	0.634	0.911	0.663	0.885	0.875	0.375	0.827	0.300	0.694	0.966	0.792
	**N**	21	21	21	20	20	20	21	21	21	19	19	19

*N* Number of analyzed individual samples, *P* P-value for the correlation, *E2* Free estradiol-17ß, *E2/T* Free estradiol-17ß / testosterone ratio, *T* Testosterone, *Rs* Spearman-rho correlation coefficient. Bold data represent significant correlations (*P* < 0.05)

### Amino acid substrate profiles of TauT and LAT1 in maternal and cord plasma, and placenta

mTORC1 and the amino acid transporters LAT1 and TauT operate as interacting regulators and mediators of amino acid transport, respectively [12, 16, 17]. Hypothesizing that the observed placental expression differences for *mTOR* mRNA, *SLC7A5* mRNA, *SLC6A6* mRNA, and LAT1 have changed the respective protein levels and/or activities and thereby the amino acid substrates of LAT1 (His, Ile, Leu, Met, Phe, Trp, Tyr, and Val) and TauT (Tau) in maternal and fetal compartments, we analyzed respective amino acid levels in the maternal plasma at week-15 and week-32 of gestation (Table 6) and in the offspring the chorionic villous portion of the placenta as well as cord plasma (Table 7). The subsequent data analyses considered: (1) differences between the n-3 LCPUFA intervention and control group (group effect), (2) differences between male and female placentas (group-independent sex effect), and (3) the interaction between group and sex (group-dependent sex effects). The following abbreviations for Tau and Trp levels in specific maternal and fetal compartments will be used in the text henceforth: P15-Tau and P15-Trp represent Tau and Trp levels in maternal plasma at week-15 of gestation (P15), respectively. P32-Tau and P32-Trp represent Tau and Trp levels in maternal blood plasma at week-32 of gestation, respectively. PL-Tau and PL-Trp represent Tau and Trp levels in the placental chorionic villous portion of the placenta, respectively. UC-Tau and UC-Trp represent Tau and Trp levels in umbilical cord blood plasma, respectively.

**Table 6:** Amino acid analysis of maternal plasma at week-15 and week-32 of gestation

**Week-32 of gestation (P32)**															
**Amino acid levels** **(μmol/L)**	**Control group**				**n-3 LCPUFA intervention group**				***P*-values regression**						
	**Con-F (*n*** **= 10)**		**Con-M (*n*** **= 9)**		**N3-F (*n*** **= 10)**		**N3-M (*n*** **= 10)**		**Treatment**	**Sex**	**Interaction**	**Con-M** **vs** **Con-F**	**N3-M** **vs** **N3-F**	**N3-F** **vs** **Con-F**	**N3-M** **vs** **Con-M**
	Median (25th - 75th)		Median (25th - 75th)		Median (25th - 75th)		Median (25th - 75th)								
**Tau**	**98.95**	**(76.60–122.08)**	**105.70**	**(90.75–118.25)**	**92.90**	**(89.18–103.93)**	**78.55**	**(70.45–88.95)**	**0.027**	0.425	0.285	0.830	0.191	0.376	**0.023**
**His**	80.70	(72.05–83.68)	74.80	(65.80–76.55)	76.60	(72.28–80.85)	74.70	(68.93–82.35)	0.979	0.650	0.433	0.387	0.829	0.603	0.561
**Val**	155.20	(140.20–176.68)	149.10	(140.45–158.75)	163.25	(132.05–179.73)	156.20	(131.55–172.65)	0.696	0.697	0.683	0.576	0.983	0.985	0.575
**Met**	9.20	(8.50–10.65)	9.20	(7.75–10.65)	8.90	(7.73–10.93)	10.25	(8.70–11.00)	0.422	0.425	0.592	0.865	0.348	0.842	0.349
**Tyr**	31.25	(29.65–35.98)	32.80	(29.60–38.45)	36.45	(33.45–39.50)	32.15	(29.23–37.08)	0.189	0.803	0.508	0.521	0.755	0.166	0.687
**Ile**	42.30	(32.90–45.23)	36.70	(33.55–46.45)	41.30	(39.18–45.33)	38.80	(34.87–43.65)	0.965	0.546	0.918	0.726	0.618	0.919	0.968
**Leu**	71.45	(66.05–75.33)	67.10	(62.60–83.05)	79.95	(72.95–82.90)	73.85	(63.98–78.45)	0.269	0.558	0.640	0.929	0.461	0.271	0.646
**Phe**	37.25	(34.65–38.95)	38.10	(33.15–39.05)	39.60	(38.58–42.08)	38.45	(35.20–40.85)	0.063	0.556	0.428	0.912	0.329	0.065	0.459
**Trp**	**35.05**	**(32.78–39.05)**	**37.40**	**(35.05–42.35)**	**42.70**	**(40.60–45.28)**	**39.85**	**(34.05–44.53)**	**0.004**	0.613	0.225	0.225	0.596	**0.004**	0.217
**Week-15 of gestation (P15 = baseline)**															
**Amino acid levels** **(μmol/L)**	**Control group**				**n-3 LCPUFA intervention group**				***P*-values regression**						
	**Con-F (*n*** **= 11)**		**Con-M (*n*** **= 9)**		**N3-F (*n*** **= 10)**		**N3-M (*n*** **= 11)**		**Sex**						
	Median (25th - 75th)		Median (25th - 75th)		Median (25th - 75th)		Median (25th - 75th)								
**Tau**	106.50	(67.60–131.10)	110.60	(103.60–142.85)	108.40	(66.45–131.03)	108.70	(92.20–142.30)	0.175						
**His**	68.80	(64.60–76.60)	69.30	(61.45–73.40)	70.95	(63.68–75.88)	62.50	(59.90–66.70)	0.059						
**Val**	168.30	(157.20–191.60)	172.40	(146.00–185.05)	184.55	(159.20–208.55)	160.30	(145.00–178.50)	0.061						
**Met**	8.70	(8.00–11.60)	9.20	(8.20–10.10)	9.80	(8.83–11.95)	10.00	(9.50–10.10)	0.612						
**Tyr**	33.30	(29.40–37.70)	36.50	(32.75–40.85)	36.10	(31.38–41.83)	30.20	(27.20–34.60)	0.178						
**Ile**	38.50	(37.30–42.90)	38.70	(37.65–40.50)	43.25	(37.88–49.55)	40.70	(37.40–45.30)	0.260						
**Leu**	80.40	(76.90–84.20)	80.40	(70.60–86.30)	86.20	(77.55–92.45)	81.20	(72.00–91.10)	0.234						
**Phe**	39.00	(36.70–43.90)	39.20	(36.70–41.10)	40.70	(38.28–46.65)	37.40	(36.30–40.40)	0.051						
**Trp**	41.30	(39.20–46.30)	44.00	(40.70–50.40)	45.40	(42.35–51.38)	42.00	(36.10–47.40)	0.274						

Amino acid data are summarized as median (25th centile - 75th centile). For the analyses at P32, the *p*-values for *Treatment* and *Sex* are from linear regression models fit to the amino acid data without a *Sex-Group Interaction* term. The *p*-values for the *Interaction* and pairwise group comparisons are from regression models including a *Sex-Group Interaction* term. The regression models were adjusted for the respective baseline (P15) levels. For P15, the *p*-values for *Treatment* are from simple linear regression models with *Treatment* as the predictor variable. Two-sided *p* < 0.05 are marked in bold

**Table 7:** Amino acid analyses of placenta and umbilical cord plasma

**Placenta**															
**Amino acid** **levels** **(μmol/g** **protein)**	**Control group**				**n-3 LCPUFA intervention group**				***P*-values regression**						
	**Con-F (*n*** **= 11)**		**Con-M (*n*** **= 8)**		**N3-F (*n*** **= 10)**		**N3-M (*n*** **= 11)**		**Treatment**	**Sex**	**Interaction**	**Con-M** **vs** **Con-F**	**N3-M** **vs** **N3-F**	**N3-F** **vs** **Con-F**	**N3-M** **vs** **Con-M**
	Median (25th - 75th)		Median (25th - 75th)		Median (25th - 75th)		Median (25th - 75th)								
**Tau**	387.01	(269.08–485.66)	317.50	(239.14–402.34)	335.87	(243.70–589.43)	277.91	(246.19–382.32)	0.919	0.266	0.962	0.430	0.432	0.916	0.973
**His**	5.42	(4.03–8.00)	4.90	(3.40–7.53)	4.60	(3.23–10.60)	4.24	(3.78–6.63)	0.605	0.508	1.000	0.644	0.636	0.706	0.729
**Val**	5.04	(4.34–7.33)	4.54	(3.49–7.14)	5.08	(3.52–9.59)	4.83	(3.35–5.40)	0.798	0.216	0.536	0.654	0.194	0.800	0.532
**Met**	2.54	(0.90–3.28)	2.03	(1.76–3.63)	3.19	(2.13–4.11)	2.85	(1.45–3.87)	0.142	0.868	0.323	0.543	0.424	0.084	0.762
**Tyr**	6.25	(5.43–8.90)	5.06	(3.88–8.75)	6.12	(3.82–12.00)	5.40	(4.10–6.03)	0.640	0.193	0.545	0.625	0.183	0.930	0.448
**Ile**	6.16	(5.66–7.64)	5.58	(4.11–7.77)	5.11	(3.95–11.33)	5.62	(3.75–6.74)	0.840	0.271	0.736	0.605	0.308	0.932	0.705
**Leu**	4.94	(4.44–7.06)	4.47	(3.36–6.09)	3.98	(3.29–8.88)	4.04	(3.13–5.44)	0.806	0.212	0.648	0.590	0.229	0.885	0.620
**Phe**	4.85	(4.27–7.17)	4.31	(3.42–5.92)	4.26	(3.24–9.41)	4.25	(3.30–5.33)	1.000	0.172	0.652	0.502	0.202	0.752	0.743
**Trp**	2.74	(2.37–3.42)	2.37	(1.68–3.30)	2.68	(1.65–4.74)	2.34	(1.86–2.94)	0.957	0.115	0.397	0.608	0.090	0.576	0.513
**Umbilical Cord plasma**															
**Amino acid levels** **(μmol/L)**	**Control group**				**n-3 LCPUFA intervention group**				***P*-values regression**						
	**Con-F (*n*** **= 8)**		**Con-M (*n*** **= 8)**		**N3-F (*n*** **= 9)**		**N3-M (*n*** **= 9)**		**Treatment**	**Sex**	**Interaction**	**Con-M** **vs** **Con-F**	**N3-M** **vs** **N3-F**	**N3-F** **vs** **Con-F**	**N3-M** **vs** **Con-M**
	Median (25th - 75th)		Median (25th - 75th)		Median (25th - 75th)		Median (25th - 75th)								
**Tau**	570.00	(384.13–1078.83)	465.15	(294.07–814.45)	**504.40**	**(360.95–586.15)**	**764.20**	**(547.35–910.70)**	0.473	0.292	0.076	0.586	**0.049**	0.451	0.077
**His**	122.10	(109.15–144.20)	125.80	(109.25–133.10)	121.10	(109.25–144.30)	125.80	(110.40–143.50)	0.969	0.686	0.980	0.764	0.777	0.965	0.992
**Val**	107.80	(91.77–121.78)	111.55	(98.60–128.73)	125.10	(90.45–135.20)	107.30	(104.45–114.70)	0.725	0.467	0.327	0.230	0.877	0.345	0.653
**Met**	38.40	(29.70–52.80)	32.20	(30.53–35.68)	31.80	(27.05–40.15)	32.80	(27.10–38.50)	0.224	0.204	0.269	0.097	0.859	0.104	0.929
**Tyr**	96.60	(75.35–120.28)	94.25	(85.60–117.60)	121.90	(91.35–132.75)	94.50	(89.30–98.00)	0.335	0.636	0.142	0.494	0.167	0.087	0.680
**Ile**	81.60	(67.50–100.15)	90.70	(66.78–110.25)	88.20	(73.15–100.20)	83.80	(73.30–97.35)	0.804	0.527	0.602	0.420	0.919	0.852	0.593
**Leu**	57.20	(50.08–62.35)	56.80	(47.38–74.45)	59.40	(48.65–66.25)	53.60	(51.05–61.20)	0.637	0.543	0.810	0.554	0.784	0.873	0.616
**Phe**	79.45	(68.53–82.78)	76.40	(72.88–85.80)	80.40	(78.65–94.75)	83.00	(77.15–87.30)	0.091	0.978	0.776	0.863	0.832	0.164	0.325
**Trp**	155.70	(123.30–181.75)	148.95	(131.80–158.00)	149.10	(135.80–209.60)	151.80	(142.65–167.20)	0.236	0.968	0.526	0.670	0.634	0.205	0.713

Amino acid data are summarized as median (25th centile - 75th centile) for the analyzed groups (Con-F and Con-M, female and male offspring of the control group, respectively; N3-F and N3-M, female and male offspring of the intervention group, respectively). The *p*-values for *Treatment* and *Sex* come from linear regression models fit to the amino acid data without a *Sex-Group* Interaction term. The *p*-values for the *Interaction* and pairwise group comparisons (Con-M vs Con-F, N3-M vs N3-F, N3-F vs Con-F, N3-M vs Con-M) are from regression models including a *Sex-Group Interaction* term. All regression models were adjusted for the respective baseline (P15) maternal plasma levels. Median (25th - 75th) values of comparisons with *p*-values < 0.05 are shown in bold

Interestingly, we found a significant intervention effect in maternal blood plasma at week-32 of gestation, with increased Trp and decreased Tau levels (Table 6). Exploratory pairwise comparisons revealed significantly higher Trp levels for intervention group mothers with female offspring than for the respective controls, and significantly lower Tau levels for intervention group mothers with male offspring than for respective controls. The regression models (with and without the interaction term) did not provide evidence of a significant difference for the amino acid substrate levels for LAT1 and TauT in the placenta or umbilical cord plasma according to group (treatment) or sex, except for UC-Tau. UC-Tau levels of male offspring in the intervention group tended to be higher than in the respective control male group and were significantly higher than in the female offspring of the intervention group (Table 7).

### Associations among placental mRNA and protein expression levels, Tau and Trp levels in maternal and fetal compartments, and their relations to offspring body composition

As already mentioned (see section *Background*) adipose tissue development can be altered by a decreased nutritional n-6/n-3 LCPUFA ratio during pregnancy and lactation, and effects of n-6 and n-3 fatty acids are mediated by altered gene expression. Furthermore, altered transfer of amino acids between the mother and fetus is one of the underlying mechanisms for fetal growth and programming of offspring obesity risk. Moreover, alterations in mTOR signaling and levels of essential amino acids, such as Trp, may affect protein synthesis and thereby muscle mass, and fetal plasma Tau levels are associated with intrauterine growth restriction. Consequently, we hypothesized that n-3 LCPUFA responsive placental expression regulations of amino acid transporters and observed altered substrate levels in the maternal and fetal circulation may be not restricted to the placenta only but can also operate in offspring adipose and muscle tissue in the presence of maternal n-3 LCPUFA supplementation. To gain early insights as to whether the observed target microRNA, mRNA and protein expression levels may have physiological effects on maternal and fetal Tau and Trp levels, we analyzed their associations with each other in an exploratory approach using linear regression analyses with unadjusted and adjusted models. In the adjusted model, the following covariates were included: (1) respective baseline levels (time point before intervention start) of Tau and Trp in maternal blood plasma at week-15 of gestation, (2) group, and (3) offspring sex. For regression models with child outcomes as dependent variables at 1 year, 3 and 5 years of age, the adjusted model included breastfeeding status at 4 months (fully breastfed or formula fed/partially breastfed) as covariate, since breast feeding is known to be associated with offspring growth [33].

For clarity and to reduce the complexity of the tables of our data in the text, the results for factors with significant associations are summarized in Tables 8–11 or mentioned in the text, whereas the respective complete datasets for all factors and associations are provided in Additional file 4: Tables S5-S8.

**Table 8:** Significant associations of placental target expression with taurine and tryptophan in fetal compartments

			Model 1		Model 2	
	Outcome variables	N	beta (95% CI)	*P*	beta (95% CI)	*P*
***mTOR***	**PL-Tau**	40	**255.92 (32.50; 479.34)**	**0.026**	**325.06 (77.33; 572.78)**	**0.012**
	UC-Tau	34	− 192.24 (− 478.48; 94.00)	0.181	−282.65 (−616.79; −51.49)	0.094
	**PL-Trp**	40	1.10 (−0.14; 2.35)	0.080	**1.46 (0.13; 2.80)**	**0.033**
	UC-Trp	34	26.65 (−14.84; 63.13)	0.200	27.86 (− 20.53; 76.24)	0.249
***SLC7A5***	PL-Tau	40	48.91 (−68.21; 166.03)	0.403	73.16 (−67.79; 214.11)	0.299
	UC-Tau	34	65.27 (−82.22; 212.27)	0.374	83.33(−91.10; 265.75)	0.325
	PL-Trp	40	0.23 (−0.41; 0.87)	0.467	0.54 (−0.16; 1.24)	0.125
	UC-Trp	34	−11.12 (−32.34; 10.10)	0.294	−17.50 (−40.79; 5.80)	0.135
**LAT1**	PL-Tau	17	26.52 (−91.42; 144.45)	0.639	56.15 (−146.28; 258.58)	0.557
	**UC-Tau**	16	194.53 (−17.01; 406.07)	0.069	**476.02 (139.63; 812.42)**	**0.010**
	PL-Trp	17	0.13 (−0.76; 1.02)	0.760	0.03 (−1.39; 1.44)	0.968
	UC-Trp	16	3.37 (−25.76; 32.50)	0.808	−30.45 (−75.29; 14.40)	0.163

Linear regression analyses: summary of significant associations of *mTOR* mRNA, *SLC7A5* mRNA and LAT1 protein with taurine (Tau) and tryptophan (Trp) is shown. ΔCq-values for mRNA and microRNA expression of respective genes were used; optical density values for LAT1 protein expression normalized to respective GAPDH protein from western blot experiments were used. Amino acid levels (μmol/g protein)-values were used for placental tissue (PL); plasma amino acid levels (μmol/L)-values were used for umbilical cord plasma (UC). Beta (95% CI) values with *P*-values < 0.05 are given in bold. *Abbreviations*: *N* Sample numbers analyzed, *P P*-value

Model 1: unadjusted

Model 2: adjusted for respective baseline levels of maternal Tau and Trp levels at week-15 of gestation (P15) before treatment, sex, and group

#### Significant associations of maternal P32-Trp with fetal UC-Trp, but no associations of maternal P32-Tau and P32-Trp with placental gene and protein expression levels

Exploring first whether changes of maternal plasma Tau or Trp levels may have influenced placental expression and fetal amino acid levels, we found that P32-Tau and P32-Trp were not significantly associated with levels of *miR-99a*, *mTOR* mRNA, *SLC6A6* mRNA, *SLC7A5* mRNA, or LAT1 protein. P32-Trp was however significantly positively associated with UC-Trp in both the unadjusted and adjusted model with beta-coefficients of 3.38 (1.11–5.64, 95% CI; *p* = 0.005) and 3.83 (0.33–7.33, 95% CI; *p* = 0.033), respectively, but P32-Tau was not (Additional file 4: Table S5).

#### Significant associations between *mTOR* mRNA and PL-Tau and PL-Trp and between LAT1 and UC-Tau

The regulation of nutrient sensing and amino acid transport is complex and regulated transcriptionally and posttranscriptionally on different levels [11, 16], whereas the involvement of microRNAs is poorly understood. Therefore, we assessed the associations of identified placental mRNA and protein levels with fetal Tau and Trp levels. Significant associations were found for *mTOR* mRNA and LAT1, but not for *miR-99a, SLC7A5* and *SLC6A6* mRNA (Table 8, Additional file 4: Table S6). As shown in Table 8, *mTOR* mRNA was significantly positively associated with PL-Tau in both the unadjusted and adjusted model, with pronounced beta-coefficients and higher values in the adjusted models. In addition, *mTOR* was positively associated with PL-Trp in the adjusted model only, showing however a very small beta-coefficient. LAT1 tended to be significantly positively associated with UC-Tau in the unadjusted model, and after adjustment became significantly positively associated with a correspondingly substantial beta-value (Table 8, Additional file 4: Table S6).

#### *mTOR* and *miR-99a* demonstrate significant associations with offspring body composition at birth, and *SLC7A5* mRNA with offspring body composition at later time points

To integrate our data on offspring body composition parameters into our study, we further analyzed whether placental target gene and protein expression or Tau and Trp levels in maternal and fetal compartments are related to offspring body composition at birth, 1 year, 3 and 5 years of age. Significant associations with offspring body composition at birth were only found for *mTOR* and *miR-99a*. *mTOR* mRNA was significantly positively associated with bodyweight/placental weight (BW/PW) ratio in the unadjusted model and tended to be significantly positively associated after adjustments. *MiR-99a* was significantly negatively associated with the BW/PW ratio in the adjusted model and tended to be significantly negatively associated with the bodyweight/body length ratio in the unadjusted model (Table 9, Additional file 4: S7A, B). Significant associations with offspring body composition at later time points were only found for *SLC7A5* mRNA (Table 10, Additional file 4: Table S7A). *SLC7A5* mRNA was significantly positively associated with both bodyweight and lean mass at 1 year, and with fat mass at 3 years of age, showing substantially increased beta-coefficient values in the unadjusted models. Furthermore, *SLC7A5* mRNA tended to be significantly associated with fat mass at 1 year of age in the unadjusted models, but not after adjustment (Table 10, Additional file 4: Table S7A, B).

**Table 9:** Significant associations of placental expression with offspring body composition at birth

			Model 1		Model 2	
	Body composition variables	N	beta (95% CI)	*P*	beta (95% CI)	*P*
***mTOR***	Placental weight (g)	41	−67.32 (− 145.04; 10.41)	0.088	−66.64 (− 155.76; 22.48)	0.138
	**BW/PW rati**o	41	**1.04 (0.10; 1.99)**	**0.032**	1.00 (−0.09; 2.08)	0.071
	Birthweight (g)	41	143.51 (−126.16; 413.19)	0.288	181.39 (− 126.51; 489.29)	0.240
	Weight/length ratio	41	1.42 (−3.56; 6.41)	0.567	2.41 (− 3.25; 8.08)	0.394
	Fat mass (g)	39	55.21 (−47.34; 157.75)	0.282	50.10 (−64.37; 164.58)	0.380
	Lean mass (g)	39	86.93 (−133.42; 307.29)	0.429	120.67 (−133.03; 374.36)	0.341
***miR-99a***	Placental weight (g)	38	10.58 (−35.86; 57.03)	0.647	25.94 (−29.17; 81.05)	0.346
	**BW/PW ratio**	38	−0.44 (−0.99; 0.12)	0.118	**−0.76 (−1.40;− 0.13)**	**0.019**
	Birthweight (g)	38	−122.37 (− 272.82; 38.08)	0.108	−144.99 (−325.89; 35.91)	0.113
	Weight/length ratio	38	−2.65 (−5.36; 0.07)	0.056	−2.84 (−6.12; 0.44)	0.088
	Fat mass (g)	36	−15.20 (77.53; 47.13)	0.623	−25.65 (−97.65; 46.35)	0.473
	Lean mass (g)	36	−95.99 (−225.10; 33.13)	0.140	−104.34 (−258.65; 49.97)	0.178

Linear regression analyses: summary of significant associations of regulated placental expression of *mTOR* mRNA and *miR-99a* with offspring body composition at birth is shown. ΔCq-values for mRNA and microRNA expression of respective genes were used. Beta (95% CI) values with *P*-values < 0.05 are given in bold. *Abbreviations*: *BW* Birthweight, *N* Sample numbers analyzed, *P P*-value, *PW* Placental weight

Model 1: unadjusted

Model 2: adjusted for respective baseline levels of maternal blood plasma Tau and Trp at week-15 of gestation (P15) before treatment, sex, and group

**Table 10:** Significant associations of *SLC7A5* mRNA with offspring body composition up 5 years

				Model 1		Model 2	
	Body composition variables		N	beta (95% CI)	*P*	beta (95% CI)	*P*
***SLC7A5***	1 year	**Weight (g)**	40	**558.89 (44.09; 1073.68)**	**0.034**	298.86 (−328.20; 925.92)	0.340
		Fat mass (g)	40	168.56 (−21.09; 358.20)	0.080	143.14 (−94.10; 380.38)	0.229
		**Lean mass (g)**	40	**390.32 (22.79; 757.87)**	**0.038**	155.72 (− 283.62; 595.07)	0.477
	3 years	Weight (g)	36	971.58 (−47.11; 1990.26)	0.061	562.71 (− 730.36; 1855.78)	0.382
		**Fat mass (g)**	22	**492.81 (10.88; 974.73)**	**0.045**	362.20 (−207.62; 932.02)	0.198
		Lean mass (g)	22	541.14 (− 537.77; 1620.05)	0.308	127.01 (− 1125.52; 1379.54)	0.833
	5 years	Weight (g)	34	1318.28 (− 256.63; 2893.18)	0.098	608.94 (− 1346.48; 2564.37)	0.529
		Fat mass (g)	23	539.54 (− 169.71; 1248.79)	0.129	437.27 (− 309.48; 1184.02)	0.234
		Lean mass (g)	23	895.84 (− 722.26; 2513.94)	0.263	166.60 (− 1605.80; 1939.00)	0.846

Linear regression analyses: summary of significant associations between regulated placental expression of *SLC7A5* mRNA and offspring body composition at 1 year, 3 and 5 years of age is shown. ΔCq-values for *SLC7A5* mRNA expression were used. Beta (95% CI) values with *P*-values < 0.05 are given in bold. *Abbreviations*: *N* Sample numbers analyzed, *P P*-value

Model 1: unadjusted

Model 2: adjusted for respective baseline levels of maternal blood plasma Tau and Trp at week-15 of gestation (P15) before treatment, sex, and group. Adjusted additionally for breastfeeding status (fully, partially breastfeeding/formula) from 1 year

#### Maternal P32-Tau and P32-Trp and fetal UC-Trp show significant associations with body composition outcomes at later time points

Surprisingly, maternal and fetal Tau and Trp levels were not significantly related to offspring body composition at birth. With regard to body composition outcomes at later time points, maternal P32-Tau and P32-Trp and fetal UC-Trp showed significant associations and respective beta-coefficient values were rather moderate for P32-Trp and small for P32-Tau and UC-Trp (Table 11). No significant associations were observed for PL-Tau, PL-Trp and UC-Tau (Additional file 4: Table S8A, B). Interestingly, P32-Trp was significantly positively associated with bodyweight at 1 year and 5 years of age, with lean mass at 1 year and 3 years of age, and with fat mass at 3 years of age in the adjusted models only (Table 11, Additional file 4: Table S8A, B). P32-Tau was significantly negatively associated with bodyweight at 1 year and 3 years of age only in the unadjusted models and with lean mass at 1 year of age in both models (Table 11, Additional file 4: Table S8A, B). Fetal UC-Trp levels were significantly positively associated with lean mass at 3 and 5 years of age in the unadjusted model, whereas after adjustments significant positive associations with lean mass and bodyweight were only found at 5 years of age.

**Table 11:** Significant associations of maternal and fetal plasma taurine and tryptophan with offspring body composition up to 5 years

	Body composition variables			Model 1		Model 2	
			N	beta (95% CI)	*P*	beta (95% CI)	*P*
**P32-Tau**	1 year	**Weight (g)**	38	**−18.34 (−33.69; −2.99)**	**0.021**	−15.03 (−33.47; − 3.41)	0.107
		Fat mass (g)	38	−3.10 (−8.96; 2.77)	0.291	−2.08 (−9.44; 5.28)	0.570
		**Lean mass (g)**	38	**−15.24 (−25.83; −4.65)**	**0.006**	**−12.95 (−25.41; −0.49)**	**0.042**
	3 years	**Weight (g)**	34	**−30.90 (−60.30; −1.46)**	**0.040**	−20.89 (−60.27; 18.49)	0.287
		Fat mass (g)	22	−8.50 (−22.44; 5.43)	0.218	−7.21 (−25.53; 11.12)	0.417
		Lean mass (g)	22	−20.72 (−49.20; 7.75)	0.145	−14.35 (−52.0; 23.30)	0.431
	5 years	Weight (g)	32	−42.86 (−95.90; 0.20)	0.051	−39.73 (−95.70; 16.23)	0.156
		Fat mass (g)	23	−14.78 (−34.58; 5.02)	0.136	−10.41 (−36.21; 15.39)	0.407
		Lean mass (g)	23	−39.44 (−82.34; 3.45)	0.070	−34.76 (−90.29; 20.76)	0.204
**P32-Trp**	1 year	**Weight (g)**	38	48.26 (−15.59; 112.11)	0.134	**86.19 (−0.30; 172.08)**	**0.049**
		Fat mass (g)	38	10.58 (−12.85; 34.02)	0.366	24.49 (−8.67; 57.64)	0.142
		**Lean mass (g)**	38	37.68 (−7.45; 82.81)	0.099	**61.70 (1.49; 121.91)**	**0.045**
	3 years	Weight (g)	34	87.12 (−36.64; 210.89)	0.161	158.70 (−9.68; 327.09)	0.064
		**Fat mass (g)**	22	53.15 (−8.32; 114.61)	0.086	**79.04 (0.22; 157.86)**	**0.049**
		**Lean mass (g)**	22	107.09 (−21.06; 235.23)	0.097	**211.89 (63.92; 359.87)**	**0.008**
	5 years	**Weight (g)**	32	105.16 (−96.10; 306.42)	0.294	**257.23 (6.30; 508.16)**	**0.045**
		Fat mass (g)	23	51.01 (−31.25; 133.26)	0.211	51.72 (−57.67; 161.11)	0.332
		Lean mass (g)	23	74.75 (−112.21; 261.70)	0.415	181.40 (− 58.58; 421.38)	0.129
**UC-Trp**	1 year	Weight (g)	33	4.74 (−3.90; 13.36)	0.272	5.90 (− 3.35; 15.14)	0.202
		Fat mass (g)	33	0.44 (−3.15; 4.03)	0.805	0.67 (− 3.23; 4.57)	0.728
		Lean mass (g)	33	4.30 (−1.59; 10.19)	0.147	5.23 (− 1.06; 11.51)	0.099
	3 years	Weight (g)	29	8.83 (−6.74; 24.40)	0.255	9.18 (−7.89; 26.26)	0.277
		Fat mass (g)	18	7.16 (−1.61; 15.93)	0.103	7.63 (−1.77; 17.03)	0.100
		**Lean mass (g)**	18	**14.68 (0.83; 28.54)**	**0.039**	15.88 (0.80; 32.55)	0.060
	5 years	**Weight (g)**	28	20.36 (−5.80; 46.52)	0.122	**27.94 (0.98; 54.90)**	**0.043**
		Fat mass (g)	20	7.33 (−5.88; 20.53)	0.259	7.98 (−5.53; 20.48)	0.226
		**Lean mass (g)**	20	**23.19 (3.11; 43.27)**	**0.026**	**28.56 (6.58; 50.54)**	**0.015**

Linear regression analyses: summary of significant associations between levels of maternal and fetal plasma taurine (Tau) and tryptophan (Trp) and offspring body composition at 1 year, 3 and 5 years of age. Amino acid levels (μmol/L)-values for maternal plasma at week-32 of gestation (P32) and umbilical cord plasma (UC) were used. Beta (95% CI) values with *P*-values *P* < 0.05 are given in bold. *Abbreviations*: *N* Sample numbers analyzed, *P P*-value

Model 1: unadjusted

Model 2: adjusted for respective baseline levels of maternal blood plasma Tau and Trp at week-15 of gestation (P15) before treatment, sex, and group. Adjusted additionally for breastfeeding status (fully, partially breastfeeding/formula) from 1 year

## Discussion

Through our initial explorative microRNA profiling of female placentas from the n-3 LCPUFA intervention and control groups we identified 25 differentially regulated microRNAs and 21 microRNAs that were only present in one or the other group. Bioinformatics analyses and predictions for microRNA binding sites on n-3 LCPUFA regulated mRNAs, previously identified by our transcriptome analyses [10], suggested interactions between *miR-99a* and its targets *mTO*R, *SLC7A5*, and *SLC6A6,* encoding the nutrient sensor mTOR and the amino acid transporters LAT1 and TauT, respectively. Subsequent validations of selected microRNAs and respective targets, using samples from larger numbers of female and male INFAT placentas, showed significant expression regulation among the selected microRNAs only for *miR-99a*. Consequently, we first focused our analyses on *miR-99a* and respective targets. Further validations revealed sexually dimorphic expression differences per se in the control group, showing higher levels for placental *miR-99a*, *mTOR* and *SLC7A5* mRNA in male offspring. In contrast, we found sex-specific n-3 LCPUFA responsive upregulation in the intervention group female placentas of *miR-99a*, *mTOR* mRNA, and *SLC7A5* mRNA and downregulation of LAT1. These sex-specific regulations apparently explain the largely balanced expression levels observed between male and female offspring placentas from the intervention group compared to the control group. In contrast, downregulation of placental *SLC6A6* mRNA was independent of sex in the intervention group. These findings are in line with our previous data on placental sexually dimorphic mRNA expression in the INFAT study subpopulation [10]. Considering sex steroid hormone-mediated gene expression regulation as a potential underlying mechanism for the observed sex-specific gene expression, only moderate n-3 LCPUFA treatment-independent correlations between E2 and *SLC7A5* mRNA in male placentas and between T and *miR-99* in female placentas were found. Furthermore, correlation analyses between *miR-99a* and its potential mRNA targets *mTOR*, *SLC7A5,* and *SLC6A6* revealed both positive and inverse interactions, pointing to a regulated expression network. With regard to amino acid level changes in maternal and cord plasma, we identified increased Trp levels for mothers with female offspring and decreased Tau levels for mothers with male offspring in the intervention group. Moreover, Tau levels in cord plasma were higher in male than female offspring in the intervention group, suggesting an inverse change for Tau in mother-male offspring pairs after n-3 LCPUFA supplementation. Surprisingly, no significant amino acid changes were observed in placentas. Regarding amino acid transporter LAT1 and TauT expression, regulation or activity, *mTOR* mRNA is associated with placental Tau and Trp levels. Furthermore, *miR-99a* and *mTOR* show negative and positive associations with offspring BW/PW ratios at birth, respectively, whereas *SLC7A5* mRNA and Tau and Trp levels in maternal and cord plasma are positively associated with bodyweight, fat mass or lean mass at 1 year, 3 or 5 years of age. Taken together, our presented data point to a placental molecular regulatory network as part of the maternal-placental crosstalk, possibly modulating and/or counteracting n-3 LCPUFA-induced changes to contribute to the stabilization of respective placental and fetal amino acid levels.

### Placental *miR-99a* and putative targets *mTOR*, *SLC7A5,* and *SLC6A6*

The presence of *miR-99a* in various human tissues, including the placenta, was reported previously by profiling specific microRNAs including *miR-99a* [47]. In the present study, *miR-99a* and its potential mRNA targets *mTOR, SLC7A5,* and *SLC6A6* were identified for the first time to be regulated in the placenta following n-3 LCPUFA supplementation. *miR-99a* can directly suppress the expression of its functional mRNA target *mTOR* in various cancer cell lines [37–39]. Interestingly, we identified a n-3 LCPUFA responsive down-regulation for *SLC6A6* mRNA that is inversely correlated with *miR-99a*. In contrast, the observed positive correlations between *miR-99a* and *mTOR* mRNA and *SLC7A5* mRNA were unexpected, given that microRNA and respective mRNA targets are in general inversely associated [21–23, 48]. Our data might however reflect increased *mTOR* and *SLC7A5* mRNA levels, which have already been dimmed to some extent by concurrent posttranscriptional counteraction through up-regulated *miR-99a*. This explanation implies that the transcriptional promoter activities of the target gene are massively higher than the respective measured mRNA levels, which is analyzable by nuclear run-on experiments [49]. Another explanation might be that the upregulation of the microRNA target gene was stimulated by the direct binding of *miR-99a* to the target gene itself, as described by Vasudevan [23]. In addition, microRNAs can also mediate translational repression or posttranslational control [42, 43, 48], which may explain decreased LAT1 expression but positive *miR-99a* association with *SLC7A5* mRNA. Therefore, the putative interaction of placental *miR-99a* with its target might be different compared to cancer cells [37–39]. Our data on sex-specific and n-3 LCPUFA-responsive expression changes and associations between *miR-99a* and target genes will give new insights into regulatory mechanisms of amino acid transport in the placenta. They are consistent with previous findings, that mTOR shows important regulatory relations to LAT1 and TauT [11, 12, 16, 17] and identifies *miR-99a* as a potential expression modulator of *SLC7A5* and *SLC6A6* during physiologically adaptive responses. Whether steroid hormones are involved in the observed sex-specific and/or n-3 LCPUFA dependent expression regulation cannot be answered with our data. However, the moderate significant positive correlations between E2 and *SLC7A5* and between T and *miR-99a* are interesting in the context of (1) amino acid transporter such as SLC7A5 protein to function upstream of mTORC1 to allow cells to sense amino acid availability and launch respective anabolic or catabolic responses [50] and (2) the reported genomic and post-translational crosstalk between estrogen receptor-α signaling und mTOR [51].

To validate our findings on cellular level, future functional analyses in vitro have to be performed using *miR-99a* and mRNA target expression systems and isolated cell types from term placentas such as syncytiotrophoblasts, cytotrophoblasts, or endothelial cells.

### Regulation of *miR-99a*, *mTOR*, *SLC7A5*, and *SLC6A6* following n-3 LCPUFA supplementation and their relation to amino acid transport

N-3 LCPUFA DHA downregulates mTORC1 activity and amino acid transport in primary human trophoblast cells [52], whereas *PPARγ* agonists, including 15d-PGJ2, can stimulate *SLC7A5* and *SLC6A6* expression in placental trophoblasts. This is consistent with placental mTORC1 signaling determining fetal growth via regulation of nutrient transporters and linking maternal nutrient availability with fetal growth [12]. The complexity of mTORC1-mediated regulation of amino acid transporters is reflected by several in vitro studies with human trophoblasts. Inhibition of mTORC1 reduces the mRNA levels of *SLC6A6* and *SLC7A5*, but discrepancies between *SLC7A5* mRNA and LAT1 protein expression were also reported [40]. Furthermore, mTORC1-regulated amino acid transport can depend on glucose and growth [53] and is modulated by the ubiquitin-proteasome system [54–56]. More recently, Lager et al. [57] observed inverse associations between DHA and placental mTOR and amino acid transporter expression and activity in syncytiotrophoblasts isolated from the placentas of n-3 LCPUFA DHA supplemented obese pregnant women. Our data are not fully in line with these findings, since we observed increased placental *mTOR* and *SLC7A5* mRNA and decreased LAT1 and *SLC6A6* mRNA following n-3 LCPUFA supplementation. These discrepancies may be explained by study design differences. In the INFAT study, only healthy mothers with non-obese pre-gravid BMI (BMI < 30) were included, n-3 LCPUFA supplements were n-3 LCPUFA DHA and EPA, and the intervention started at 15 weeks’ gestation and continued until 4 months postpartum. In comparison, Lager et al. [57] analyzed syncytiotrophoblasts from term placentas of obese pregnant women supplemented with DHA from week-26 of gestation to term. Furthermore, the complexity of data on various regulatory levels and differences in n-3 LCPUFA effects on mTORC1 activation and amino acid transport, as shown by in vitro and in vivo studies using different cell-types and species, should be taken into account. For example, mTORC1 activation decreased in various cancer cell lines following n-3 LCPUFA treatment [58–60], but strong mTORC1 activation was observed in muscle tissue from n-3 PUFA supplemented healthy humans [61, 62] or steers [63] only after amino acid and insulin stimulation. Consequently, the n-3 LCPUFA effects on mTOR and amino acid transport seem (1) to depend on the type of n-3 LCPUFA and respective metabolites, (2) to be different in healthy and diseased conditions, (3) to depend on the cell-type and/or organ, and (4) to be sensitive to the time-window of intervention.

### Changes for Trp and Tau levels in maternal and fetal circulation

Due to the lack of primary cells from the placental specimens in our study, the observed placental n-3 LCPUFA effects could not be further extended in vitro. However, we analyzed amino acid levels in maternal and fetal compartments, demonstrating lower Tau and higher Trp levels in maternal plasma following the n-3 LCPUFA intervention, while placental Tau and Trp levels remained unchanged, and Tau levels were increased in cord plasma of male offspring. Tau has antioxidant and cytoprotective properties, plays important roles in syncytiotrophoblast development and function, and fetal growth and organ development. Fetal plasma Tau levels are also associated with intrauterine growth restriction [64–66]. Human TauT is located at the maternal facing brush-border membrane of syncytiotrophoblasts [64, 67, 68] and is assumed to be essential for fetal Tau supply from the maternal circulation since the fetus cannot synthesize Tau [69, 70]. Interestingly Holm et al. [71] recently identified the placental expression of the Tau biosynthetic enzyme cysteine sulfinic acid decarboxylase and placental bilateral Tau release to the maternal and fetal compartment. Furthermore, DHA can regulate the expression of cystathionine-γ-lyase, which is rate-limiting for homocysteine degradation in the transsulfuration pathway to Tau [72] and n-3 LCPUFA consumption decreases plasma homocysteine levels [73]. This suggests that n-3 LCPUFA intervention can potentially impact Tau biosynthesis by altering Tau levels in the maternal tissue, the circulation and the placenta. Given the potential bilateral Tau release from the placenta and its unknown regulation [69], we hypothesize that both the observed reduced maternal plasma Tau levels and the increased Tau levels in cord plasma might reflect lower Tau biosynthetic activity by maternal organs, such as the liver, and consequently increased placental Tau biosynthetic activity and efflux to the fetal circulation. This may be accomplished by downregulating *SLC6A6* gene expression and Tau uptake activity at the maternal side of the syntiotrophoblast. These assumptions are clearly speculative and certainly need more research and clarification by further studies, since the knowledge about Tau transfer and regulation at the maternal-fetal interface from human in vivo studies is still scarce.

Trp as an essential amino acid is important for fetal growth and development, but also for serotonin production. Its signaling pathways regulate fetal rejection suppression and feeding and satiety [74]. Furthermore, the Trp pathway can target placental antioxidant capacity, including mTORC1 [75]. Interestingly, n-3 LCPUFA EPA reduces indoleamine 2,3–dioxygenase, which catalyzes Trp to kynurenine conversion in tumor cells [76]. In this context, it will be of interest for future investigations to assess whether elevated maternal Trp plasma levels are due to changes in Trp metabolism and flux following an n-3 LCPUFA intervention and if the observed placental LAT1 decrease reflects the accompanying molecular response.

Altogether, it is tempting to speculate that the observed placental expression changes and miRNA-mRNA interactions in the intervention group may represent the molecular and physiological response of the placental nutrient sensing system (1) to n-3 LCPUFA-induced maternal physiological changes affecting maternal Tau and Trp levels, and/or (2) to the broad impact of n-3 LCPUFA on placental gene expression, thereby counteracting changes and contributing to placental homeostasis of placental and fetal amino acid levels. Our findings might have revealed new aspects and/or a part of a regulatory network which is vital to suffice the fetal demands for normal growth and to stablize maternal and fetal interactions for normal pregnancy, and consequently protect the developing fetus from adverse effects.

### Associations among gene and protein expression, amino acid levels, and offspring body composition

Altered transfer of amino acids between the mother and fetus is one of the underlying mechanisms for fetal growth and programming of offspring obesity risk [3, 4]. Therefore, we hypothesized that our identified targets may be not only the n-3 LCPUFA responsive in the placenta but also in the offspring adipose and muscle tissues. In this context, studies analyzing human placentas in relation to intrauterine growth restriction, preterm birth, and SGA (small for gestational age) have shown positive associations of low placental levels of PPARγ, LAT1, or TauT with birthweight, indicating their involvement in the control of fetal growth [11, 65–68]. A key role in placental adaptation and mediation of programming effects was proposed for placental nutrient sensing pathways, including mTOR [16, 77]. Regarding growth regulation of adipose tissue and muscle, mT*OR*, *SLC7A5* and *SLC6A6* are expressed also in adipocytes [78–80] and n-3 LCPUFA affect muscle metabolism of healthy humans [61, 62] and steers [63]. For Trp, tryptophan metabolite 5-hydroxytryptamine (serotonin) can regulate white and brown adipose tissue function, including adaptive thermogenesis in mice [81]. Tau effectively inhibits adipocyte formation in a human adipose-derived stem cell model, whereas TauT substrates hypotaurine and β-alanine promote adipogenesis [82]. Moreover, long-term Tau supplementation in mildly obese mice can inhibit adipogenesis in muscle and white adipose tissue, but not brown adipose tissue [83]. However, whether these anti-obesity effects are also present in humans is not known and respective clinical trials are needed. In this study, our data on associations among *miR-99a* and its putative targets suggest that *miR-99a* can target their expression on different levels. With regard to offspring body compositions, only *miR-99* and *mTOR* mRNA showed positive and negative associations with offspring BW/PW ratio at birth, respectively. *SCL7A5* mRNA showed significant positive associations and substantial effect size with weight and lean mass at 1 year and 3 years of age, respectively. This is interesting, since SLC7A5 protein plays an essential role sustaining mTORC1 activity that is a master regulator of cell size and tissue mass [50]. Moreover, concerning fetal and offspring growth, placental *SLC7A5* mRNA and LAT1 are significantly associated with cord plasma Trp and Tau in the present study. Surprisingly, the effect size of associations between maternal Tau and Trp and fetal Trp levels with respective offspring body composition values were small, except for the link between maternal Trp and bodyweight at 5 years of age. Since significant associations were often only observed either in the unadjusted or the adjusted model, it will be interesting to find out which of the various covariates are involved. It is important to note that offspring bodyweight in the intervention and control groups did not differ significantly from 1 year onwards, neither in the entire INFAT study population nor the INFAT subgroup in the present and previous studies [31–34, 84]. The present data indicate that the observed sex-specific expression changes and Tau and Trp level alterations and their respective associations with offspring body composition need more detailed analyses to find out exactly which sex and sex-group interactions significantly matter. This certainly requires data and analyses from a larger number of mother-offspring pairs and respective placenta and plasma samples.

### Strength and limitations

Several limitations warrant mentioning. First, our explorative approach was limited because of the small numbers of placenta pools and only female placentas were analyzed in our initial microRNA profiling experiments. This design cannot provide complete group and sex-specific representation and therefore represents an initial limitation of our study. Second, no functional validation of the identified putative miRNA-mRNA interactions was performed in vitro. Third, due to the limitations of our protein analyses and the lack of isolated cells from sampled placental tissue for in vitro studies, we could not assess experimentally mTOR and TauT protein expression, mTORC1 activation and signaling, and amino acid transporter activities. Finally, our association analyses were limited because maternal blood samples were not collected at birth. Consequently, there was no data available on maternal plasma amino acid levels.

The strength of our study compared to previous in vitro-based studies was that we investigated the chorionic villous tissue of the term placenta. This includes the major placental cell types for transport, such as syncytiotrophoblasts and endothelial cells, which were exposed to the physiological maternal-fetal environment in vivo, including all factors, such as hormones, growth factors, and nutrients. Therefore, our data may represent the complex in vivo situation, which can also explain some of the discrepancies between our data and other studies. However, both in vitro and in vivo studies are important to gain new insights and develop working hypotheses, not only to promote a better understanding of placental physiology and pathology but also to investigate underlying molecular mechanisms and cross-talk. Another strength of our integrative analyses was that relatively large sample numbers were analyzed per group for molecular analyses and sex-specific biological validations. Moreover, several maternal and fetal tissues were investigated, including samples from (1) maternal plasma during pregnancy and (2) placentas and cord plasma from spontaneous birth, to reveal associations with offspring body composition and explore potential fetal and offspring adaptation and programming effects from birth up to 5 years of age. Furthermore, INFAT mother-offspring pairs were extensively characterized and their excellent compliance to the intervention was previously demonstrated by the LCPUFA profile observed in maternal and cord plasma and red blood cells [31].

## Conclusions

Our exploratory study revealed *miR-99a* as a putative novel regulator in the complex molecular network of placental *mTOR* expression and amino acid transport. The findings described herein may reflect gene expression responses and potential microRNA-mRNA interactions in the placenta to both the altered Tau and Trp levels in the maternal and fetal circulation and the broad placental transcriptional changes following an n-3 LCPUFA intervention. These observed alterations and interactions certainly represent only a fraction of possible gene regulatory activities in the presence of enhanced maternal n-3 LCPUFA with or without physiological consequences, but they may have contributed to maintaining amino acid homeostasis in placental and fetal circulation, thereby protecting the fetus from potential adverse effects. Further validations are warranted to validate the putative interactions of placental *miR-99a* with *mTOR, SLC7A5* and *SLC6A6* mRNA by functional experiments and to pursue the analyses of placental *mir-99a* and *SLC7A5* mRNA and maternal and umbilical cord plasma Tau and Trp levels as potential predictive biomarkers for offspring body composition in larger studies. The underlying cause-and-effect relationship of maternal and fetal responses and alterations as a result of nutritional and environmental changes or interventions and their relationships to offspring body composition, including potential fetal and metabolic programming effects, are elusive and certainly need more research efforts.

## Methods

### Ethics statement and INFAT study, and INFAT study subpopulation

The presented study is a secondary analysis from the INFAT study [7, 31] using biosamples and data from a defined subpopulation of the INFAT cohort. The characteristics of the subpopulation and respective placental mRNA expression regulation in the presence of enhanced maternal n-3 LCPUFAs were already described in our previous report [10]. In the present study, we extended the analyses of this subpopulation using respective biosamples and data as described below.

The INFAT study addressed as its primary endpoint whether a reduced n-6/n-3 LCPUFA ratio in maternal nutrition during pregnancy and lactation might represent a strategy to reduce offspring adipose tissue growth [7, 31]. The study was conducted in accordance with the Declaration of Helsinki, and the protocol was approved by the Ethics Committee of the Technical University of Munich, Germany (1479/06/2006/2/21). All participants gave their written informed consent for inclusion prior to participation. All human materials used were obtained from study participants by written informed consent provided by the parents [7, 31]. The study protocol is registered at the ClinicalTrials.gov Protocol Registration System with the number ID NCT00362089 (http://clinicaltrials.gov/ct2/show/NCT00362089). The INFAT study was an open-label, monocenter, prospective, randomized, controlled dietary intervention trial in a 2-arm parallel group design as described in detail by Hauner et al. [7, 31]. In brief, in the INFAT study, 208 women of Western European descent with a BMI between 18 and 30 without high-risk pregnancies or prior n-3 fatty acid supplementation were included before week 15 of gestation and randomly assigned to a control or n-3 LCPUFA intervention group. Both groups were advised to follow a healthy diet during pregnancy. The women in the intervention group were additionally advised to decrease their dietary n-6/n-3 LCPUFA ratio to about 3:1 by reducing arachidonic acid intake to 50–90 mg per day by decreasing their intake of meat and other animal products, and were instructed to take a daily n-3 LCPUFA supplement (1020 mg DHA and 180 mg EPA). Healthy term-born offspring (between week 37 and week 42 of gestation) were included for further analyses. Birthweight and length, gestational age, mode of delivery and sex of the newborn were recorded from maternal obstetric records. Anthropometric measurements (e.g. bodyweight/length ratio, bodyweight/placental weight ratio) were collected at birth and post partum at 3–5 days, 6 weeks, 20 weeks and annually thereafter from 1 year up to 5 years (i.e. bodyweight, body height, skin folds). Fat and lean mass were calculated as reported previously [7, 31]. Excellent compliance has been already demonstrated for the entire INFAT study population [31], including the women in the INFAT subpopulation of our previous [10] and present placental expression analyses. The selection of the subpopulation was determined by minimizing the influences of birth mode and consideration of the use of analgesics or anaesthetics on placental gene expression as described [10]. Therefore, all analyzed placentas were derived from offspring of mothers with healthy pregnancies, and mothers and offspring with pregnancy complications were excluded. These offspring were born by vaginal delivery and considered as appropriate for gestational age (10th - 90th percentile of birthweight). For the selection of the INFAT study subpopulation, the main criteria were controlled sampling of placenta and cord blood from female and male offspring for subsequent analyses, together with the data availability of the mother-offspring pairs. Biosamples of both offspring sex were important to assess sex-specific biological processes in our studies. Data from defined study participants and their offspring (female, *n* = 21; male, *n* = 20) were divided into the control group (Con, n = 20) and the n-3 LCPUFA intervention group (N3, n = 21). The main clinical characteristics of INFAT study subpopulation mothers and offspring were presented previously [10] and in the present study in Additional file 1: Table S1. There were no significant differences between the groups in the investigated offspring growth parameters at birth and up to 5 years of age, consistent with the data for the respective whole INFAT cohort as previously reported [10, 31–34, 84].

### Placental and cord blood sampling

Placenta samples were dissected from four quadrants according to a standardized sampling protocol [10]. The chorionic villous portion of each placenta, obtained by removing maternal basal and fetal chorionic plates was immediately snap-frozen. Maternal blood at weeks 15 and 32 of gestation and cord blood was collected into EDTA tubes and centrifuged at 4 °C with 2000 g for 10 min. Plasma and placenta samples were stored at − 80 °C until further processing.

### Extraction of total RNA and total RNA containing small RNA

For extraction of total RNA or total RNA containing small RNA from placental tissue (chorionic villous portion), the *TRIzol* method was combined with the *midi RNeasy Kit* (Qiagen, Hilden, Germany) or the *mirPremier microRNA isolation Kit* (Sigma-Aldrich, Taufkirchen, Germany), respectively. For each placenta, equal amounts of the four placental quadrant samples were first homogenized separately in *TRIzol Reagent* (Invitrogen, Darmstadt, Germany). Afterwards, for each offspring, equal parts of the respective single quadrant *TRIzol* homogenates were combined to prepare representative and defined starting placental *TRIzol* homogenate for further RNA isolations for matched mRNA and microRNA analyses. From starting placental *TRIzol* homogenates respective total RNA and total RNA containing small RNA were extracted and subsequently quantified with the *NanoDrop 1000 Spectrophotometer* (Peqlab, Erlangen, Germany). RNA samples with RNA integrity numbers > 5 (*Agilent Bioanalyzer 2100*; Agilent Technologies, Böblingen, Germany) were included in further experiments.

### Explorative microRNA profiling

Considering our previous data showing mRNA expression changes predominantly in female intervention placentas [10], we restricted our microRNA profiling experiments first to female placental specimens of the INFAT study subpopulation, which we have already analyzed previously in our mRNA transcriptome analyses [10]. Equal amounts of total RNA containing small RNA from three respective female placentas of the control (Con, *n* = 3) and the n-3 LCPUFA intervention group (N3, n = 3) were pooled for further cDNA synthesis and amplification (one pool per group). Group-specific pools were analyzed in parallel by *Applied Biosystems TaqMan low density human microRNA assay*. All reagents and kits applied were obtained from Applied Biosystems (Darmstadt, Germany) and used according to the manufacturer’s protocol. In brief, 350 ng of pooled total RNA containing small RNA was reverse-transcribed with *TaqMan MicroRNA Reverse Transcription Kit* using *Megaplex RT primer*. Obtained cDNA was amplified *with TaqMan PreAmp Primers* and *TaqMan PreAmp Master Mix* for twelve cycles, and analyzed with *TaqMan low density human microRNA assays v2.0* and *TaqMan Universal PCR Master Mix*, *No AmpErase UNG* on the *7900HT Fast Real-Time PCR System* to assess the profile of 754 human microRNAs including endogenous and negative controls. Data were read out by the *SDS* (Sequence Detection System) *Software v2.4*. Statistical calculations were conducted in *R* (version 2.11.1). Log-fold changes were calculated as following using the term [Cq (N3) – Cq (Con)] and normalized with a cyclic *loess* procedure [85–87], separately for microRNAs detected on plate A and plate B. For the calculation of putative differentially expressed microRNAs an adaptive cut-off was determined by polynomial quantile regression (quadratic model) [88]. MicroRNAs with a log (FC) below the 0.05 or above the 0.95 quantile of the cut-off were regarded as suitable targets for further validation. The microarray data were deposited in NCBI’s Gene Expression Omnibus [89] and are accessible through GEO Series accession number GSE148326 (https://www.ncbi.nlm.nih.gov/geo/query/acc.cgi?acc=GSE148326).

### Analysis of microRNA binding sites in significantly regulated genes identified by microarray analysis

A bioinformatics approach with the *Diana mirExTra* web server application [90] was applied to select genes with significant differential placental mRNA expression, described in our previous report on mRNA microarray analyses [10], which might be predicted targets of regulated microRNAs obtained by explorative profiling. The placental specimens analyzed in the present microRNA array analyses were also included in our previous mRNA microarray analyses. The input gene list included all significantly regulated genes from three microarray datasets examined for the effect of n-3 LCPUFA intervention (intervention versus control group were analyzed separately in female and male placentas and adjusted for offspring sex; deposited in NCBI’s Gene Expression Omnibus [89] and accessible through GEO Series accession number GSE53291 (http://www.ncbi.nlm.nih.gov/geo/query/acc.cgi?acc=GSE53291). A microRNA filter was used containing microRNAs which were putatively regulated by the n-3 LCPUFA intervention in female placentas. The option ‘conservation between human and mouse’ was not applied. MicroRNAs with overrepresented binding sites in the significantly regulated genes were selected for further analysis [90].

### Gene expression analysis

Reverse transcription realtime quantitative PCR (RT-qPCR) experiments were conducted with 10 ng of total RNA for cDNA synthesis followed by realtime quantitative PCR (qPCR) using the *QuantiTect SYBR Green RT-PCR Kit* (Qiagen, Hilden, Germany) in combination with self-designed intron-spanning primer pairs or validated *QuantiTect primer assays* (Qiagen, Hilden, Germany). The primers, which were used, are listed in Additional file 5: Table S9. The cycling conditions were: 1 cycle at 50 °C for 30 min, 1 cycle at 95 °C for 15 min, 40 cycles at 95 °C for 15 s / 60 °C (or as stated in Additional file 5: Table S9) for 30 s / 72 °C for 30 s, and a terminal melting curve. Reactions were controlled by ‘no RT’ and ‘no template’ controls. Relative gene expression levels were calculated by the 2-ΔΔCt method, including normalization to the genes for *ACTB*, *POLR2a*, *B2M* and *TOP1* [91].

### MicroRNA expression analysis

As described above, microRNAs with potential binding-sites on mRNA of genes identified in our previous mRNA microarray datasets were selected for qPCR validation using RNA samples from female and male offspring placentas (*n* = 41) of the INFAT study subpopulation [10]. Oligonucleotides used for qPCR are described in Additional file 5: Table S9. 10 ng of total RNA containing small RNAs was first reversely transcribed with *TaqMan MicroRNA Reverse Transcription Kit* and analyzed by qPCR with *TaqMan Universal PCR Master Mix*, *No AmpErase UNG* (Applied Biosystems, Darmstadt, Germany) according to the manufacturer’s protocol. The cycling conditions were: 1 cycle at 95 °C for 10 min and 40 cycles at 95 °C for 15 s followed by 60 °C for 1 min. Reactions were controlled by ‘no RT’ and ‘no template’ controls. Relative gene expression levels were calculated by the 2-ΔΔCt method, including normalization to the geometric mean of three well-controlled reference genes (*RNU24*, *RNU6b*, *miR-26b*) [91].

### Extraction of protein and western blot analysis

All chemicals were obtained from Sigma Aldrich Chemie (Taufkirchen, Germany) unless stated otherwise. Equal amounts of grinded placental tissue from four-quadrant tissue samples per offspring, chosen also for respective RNA isolation as mentioned above, were homogenized in 5 μL/mg radioimmuno-precipitation assay buffer (RIPA: 150 mM NaCl, 50 mM Tris/HCl pH 8.0, 1 mM EDTA pH 8.0, 1% Nonidet-P40, 0.2% SDS, 0.25% sodium deoxycholate) supplemented with 1% protease inhibitor cocktail. A 10% SDS-polyacrylamide gel was used to separate 50 μg of denatured protein, which was subsequently transferred onto nitrocellulose membrane. The membrane was incubated with primary antibodies goat anti-human LAT1 (dilution 1:1000; Sigma Aldrich, Taufkirchen, Germany) and mouse anti-GAPDH (dilution 1:4000; Ambion Inc. / Life Technologies, Darmstadt, Germany) overnight at 4 °C. Primary antibodies specific for LAT1 and GAPDH were detected using as secondary antibodies donkey anti-goat antibody conjugated with IRDye 800CW and goat anti-mouse antibody conjugated with IRDye 680RD (1:10,000 each; LI-COR Biosciences GmbH, Bad Homburg, Germany), respectively, by incubation for 1 h at room temperature. Protein band intensities were quantified using the *LI-COR ODYSSEY* infrared imaging system and software (LI-COR Biosciences GmbH, Bad Homburg, Germany).

### Amino acid analysis

Measurements of amino acid levels in placental chorionic villous fraction and plasma were conducted as previously described [82]. Instead of the *iTRAQ Reagent Kit* used in the publication, the amino acid levels were measured with the *aTRAQ Reagent Kit 200 Assay* (ABSciex, Foster City, USA) in combination with liquid chromatography-tandem mass spectrometry (LC-MS/MS). In brief, ground placental villous tissue of four quadrants per placenta (100 mg) were each dissolved in 150 μL MeOH/H_2_O (50:50 v/v)/mg. After mixing and centrifugation (10,000 x g, 10 min, 4 °C), the four supernatants from one placenta were pooled in equal parts. Sample preparation was done using 40 μL of the supernatant of the pools or 40 μL of plasma according to the manufacturer’s instructions [92]. Mass analysis was performed using the *3200QTRAP LC/MS/MS* (Applied Biosystems, Foster City, USA). For quantification the *Analysist 1.5 Software* (Applied Biosystems, Foster City, USA) was used. Placental amino acids were normalized to the protein concentration, measured by Bradford assay.

### Statistical analysis

If not stated otherwise, participants were separated according to control (Con) or n-3 LCPUFA intervention (N3) and offspring sex in four groups: control group, male (Con-M) and female (Con-F) offspring; intervention group, male (N3-M) and female (N3-F) offspring. For analyses of gene and protein expression data, linear regression models were fit to assess differences between intervention groups and offspring sex. Regression models were fit with and without a sex-group interaction term to assess if the effect of the intervention differed according to sex. From the interaction models, exploratory pairwise group comparisons were made. For the analyses of amino acid data in maternal plasma at week-32 of gestation, placenta, and umbilical cord plasma, similar linear regression models with and without a sex-group interaction were fitted. These regression models also included the amino acid concentration in the maternal plasma at week-15 of gestation (before the intervention was administered), and the amino acid levels were log-transformed due to skewness.

For bivariate correlation analysis of expression data, non-parametric Spearman-rho correlation coefficient (Rs) and two-sided significance were calculated with IBM SPSS Statistics 19 (IBM Deutschland GmbH, Ehningen, Germany). Correlation coefficients between 0.0 and < 0.4, 0.4 and < 0.7, and, 0.7 and 1.0 were considered as weak, moderate and strong correlations, respectively.

We took an exploratory approach when investigating associations between various investigated variables in unadjusted (Model 1) and adjusted (Model 2) models. In the adjusted models, respective baseline levels of taurine (Tau) and tryptophan (Trp) at week-15 of gestation, sex and group were included as confounders. For regression models with child outcomes as dependent variables at 1 year, 3 and 5 years of age, all adjusted models included breastfeeding status at 4 months (fully breastfed or formula fed/partially breastfed) as a confounder. Linear regression models were performed with *SPSS version 25* (IBM, New York, NY, USA). A 2-sided *P-*value ≤0.05 was considered statistically significant. No corrections were made for multiple comparisons.

## Supplementary Information

**Additional file 1: Table S1.** Clinical characteristics and LCPUFA biomarker of mothers and offspring representing the INFAT study subpopulation of the previous [10] and present analyses.

**Additional file 2: Table S2.** Summary of the explorative microRNA profiling of female offspring placentas. **Table S3.** List of expressed *C19MC* microRNAs identified by microRNA profiling of female offspring placentas.

**Additional file 3: Figure S1.** qPCR validation data of selected placental target microRNAs. Scatter plots representing data shown in Table 3. **Figure S2.** qPCR validation data of selected placental target mRNAs. Scatter plots representing data shown in Table 3. **Figure S3.** Western blot composite of placental LAT1 and GAPDH expression and respective images of the original nitrocellulose membrane after successive indirect immunostainings and detections.

**Additional file 4: Table S4.** Summary of data from Sedlmeier et al. [10] on placental free estradiol-17ß (E2) and testosterone (T) levels, and E2/T ratios. **Table S5.** Associations of maternal plasma taurine and tryptophan at week-32 of gestation with placental expression and fetal taurine and tryptophan. **Table S6.** Associations of regulated placental gene and protein expression levels with taurine and tryptophan levels in fetal compartments. **Table S7A.** Model 1: unadjusted associations of regulated placental gene and protein expression levels with offspring body composition. **Table S7B.** Model 2: adjusted associations of regulated placental gene and protein expression levels with offspring body composition. **Table S8A.** Model 1: unadjusted associations of levels of taurine and tryptophan in placental tissue and cord and maternal plasma with offspring body composition. **Table S8B.** Model 2: adjusted associations of levels of taurine and tryptophan in placental tissue and cord and maternal plasma with offspring body composition.

**Additional file 5: Table S9.** Oligonucleotides for RT-qPCR.

## Data Availability

The datasets of microarray experiments for mRNA expression and microRNA expression analyses generated and/or analysed during the current study have been deposited in the NCBI’s Gene Expression Omnibus repository under the GEO Series accession numbers GSE53291 (http://www.ncbi.nlm.nih.gov/geo/query/acc.cgi?acc=GSE53291) and GSE148326. (https://www.ncbi.nlm.nih.gov/geo/query/acc.cgi?acc=GSE148326), respectively. Further datasets used and/or analyzed during the current study, which are not included in this article are available from the corresponding author on reasonable request.

## Abbreviations

AA: Arachidonic acid
Con: Control group
Con-F: Female offspring of the control group
Con-M: Male offspring of the control group
BW/PW ratio: Bodyweight-to-placental weight ratio
Cq: Quantification cycle
GAPDH: Glyceraldehyde-3-phosphate dehydrogenase
GLUT-4: Glucose transporter type 4
INFAT: Impact of nutritional fatty acids during pregnancy and lactation on early adipose tissue development
LCPUFA: Long-chain polyunsaturated fatty acids
DHA: Docosahexaenoic acid
EPA: Eicosapentaenoic acid
LAT1: L-type amino acid transporter 1
mTOR: Mechanistic target of rapamycin
mTORC: Mechanistic target of rapamycin complex
NFκB: Nuclear factor ‘kappa-light-chain-enhancer’ of activated B-cells
N3: n-3 LCPUFA intervention group
N3-F: Female offspring of the n-3 LCPUFA intervention group
N3-M: Male offspring of the n-3 LCPUFA intervention group
PPAR: Peroxisome proliferator-activated receptor
RIPA: Radio immuno-precipitation assay buffer
RT-qPCR: Reverse transcription real time quantitative PCR
SREBP: *S*terol regulatory element-binding protein
SCL2A4: Solute carrier family 2 member 4
SLC6A6: Solute carrier family 6 member 6
SLC7A5: Solute carrier family 7 member 5
TauT: Taurine transporter
UC: Umbilical cord
vs: Versus
3′-UTR: 3′-Untranslated region
5′-UTR: 5′-Untranslated region
15α-PGJ2: 15-deoxy-Δ^12,14^-prostaglandin J_2_
