# Supplementary material for: Fetal sex modulates placental microRNA expression, potential microRNA-mRNA interactions, and levels of amino acid transporter expression and substrates: INFAT study subpopulation analysis of n-3 LCPUFA intervention during pregnancy and associations with offspring body composition
Source: BMC Mol Cell Biol. 2021 Mar 3;22:15. doi: 10.1186/s12860-021-00345-x (PMC7931339; doi:10.1186/s12860-021-00345-x)
Supplement: Supplementary file 1 — Additional file 1: Table S1. Clinical characteristics and LCPUFA biomarker of mothers and offspring representing the INFAT study subpopulation of the previous [10] and present analyses. [file 12860_2021_345_MOESM1_ESM.pdf]

Additional file 1

Table S1 Clinical characteristics and LCPUFA biomarker of INFAT subgroup mothers and offspring taken from Sedlmeier et al [10]

		Male offspring (M) N	Male offspring (M) Mean ± SD / Median (IQR)	Female offspring (F) N	Female offspring (F) Mean ± SD / Median (IQR)	Con-M vs Con-F	P* N3-M vs N3-F	P# N3-F vs Con-F	P*# N3-M vs Con-M
**Maternal characteristics**									
Maternal age (years)	Con	9	33.6 ± 3.4	11	32.0 ± 4.3		0.446	0.257	0.948
	N3	11	32.6 ± 5.1	10	30.8 ± 5.1				
Primiparae‡	Con	9	44.4% n 4	11	45.5% n 5	Chi-Square test 0.880			
	N3	11	54.5% n 6	10	60.0% n 6				
Weight before pregnancy (kg)	Con	9	60.9 ± 8.1	11	63.6 ± 8.2		0.941	0.061	0.415
	N3	11	59.1 ± 6.4	10	65.7 ± 7.9				
BMI before pregnancy (kg/m^2)†	Con	9	21.0 (19.2-24.0)	11	22.7 (20.9-25.5)		0.652	0.080	0.962
	N3	11	20.8 (20.1-22.3)	10	21.9 (20.7-24.0)				
Gestational weight gain[1] (kg)	Con	9	16.1 ± 3.8	11	16.6 ± 6.5		0.641	0.506	0.715
	N3	11	14.9 ± 3.7	10	16.4 ± 3.5				
**Offspring characteristics**									
Gestational age (d)	Con	9	279.4 ± 8.7	11	282.6 ± 4.8		0.811	0.649	0.309
	N3	11	281.1 ± 5.4	10	279.9 ± 7.5				
Placental weight (g)	Con	9	551.0 ± 94.4	11	552.2 ± 70.0		0.469	0.859	0.896
	N3	11	529.0 ± 99.9	10	536.9 ± 51.8				
Birthweight (g)	Con	9	3487. ± 280.8	11	3544.6 ± 298.0		0.993	0.922	0.577
	N3	11	3536. ± 167.8	10	3496.5 ± 338.0				
Bodyweight (g) 1 year	Con	8	9636. ± 1073.7	11	9058.2 ± 922.4		0.365	0.184	0.745
	N3	11	9839. ± 1259.4	10	9486.0 ± 1020.7				
Birthweight percentile†	Con	9	48.0 (29.5-54.0)	11	54.0 (28.0-80.0)		0.857	0.210	0.900
	N3	11	41.0 (32.0-50.0)	10	57.5 (32.8-82.3)				
Birthheight (cm)	Con	9	52.2 ± 1.6	11	51.4 ± 1.4		0.495	0.469	0.350
	N3	11	52.1 ± 1.7	10	52.2 ± 1.8				
Birth head circumference (cm)	Con	9	35.6 ± 1.1	11	35.0 ± 0.8		0.312	0.064	0.914
	N3	11	35.3 ± 0.9	10	34.7 ± 1.1				
Birthweight / length (g/cm)	Con	9	66.9 ± 6.2	11	69.0 ± 5.3		0.749	0.711	0.330
	N3	11	67.9 ± 2.5	10	66.9 ± 5.6				
Birthweight / placenta weight†	Con	9	6.4 (6.0-6.9)	11	6.5 (5.9-6.9)		0.540	0.713	0.610
	N3	11	6.9 (5.5-7.6)	10	6.6 (5.9-7.0)				
**LCPUFA biomarker**									
n6/n3 LCPUFA ratio[2] Mat. RBC-P15	Con	9	2.64 (2.33-3.04)	11	2.45 (2.26-3.13)		0.276	0.301	0.136
	N3	11	2.14 (1.84-2.67)	10	2.74 (2.33-2.93)				
n6/n3 LCPUFA ratio[2] Mat. RBC-P32	Con	9	2.32 (1.91-2.69)	11	2.86 (2.20-4.64)		**< 0.001**	0.160	0.650
	N3	11	1.32 (1.07-1.99)	10	1.46 (1.30-1.74)	0.195	0.488	**<0.001**	**0.001**
n6/n3 LCPUFA ratio[2] UC RBC†	Con	8	4.90 (4.03-6.22)	9	5.39 (4.07-7.39)		**0.005**	0.106	0.291
	N3	9	2.62 (2.45-3.59)	8	3.88 (3.18-5.16)	0.680	0.062	0.172	**0.007**

Data from the control (Con) and n-3 LCPUFA intervention (N3) group are presented as N, mean ± SD or percentage for each of the four analysis groups related to the offspring or respective mothers (Con-M, control male offspring; Con-F, control female offspring; N3-M, n-3 LCPUFA intervention male offspring; N3-F, n-3 LCPUFA intervention female offspring). For quantitative variables the corresponding p-values are calculated with two-way ANOVA (P*, P#, P*#). †Not normal distributed variables or variables that violated homoscedasticity were presented as median (interquartile range IQR = 25th - 75th percentile). Their corresponding p-values were calculated with two-way ANOVA on ranks (P*, P#, P*#). In case of a significant two-way ANOVA *p*-value, *post hoc* tests (grey shaded) were conducted with Holm-Sidak test to adjust the significance level (Con-M vs Con-F, N3-M vs N3-F, N3-F vs Con-F and N3-M vs Con-M). ‡ For qualitative variables the Chi-Square test was used. *P*-values < 0.05 were considered as significant and marked bold; *P** < 0.05 significant difference between the n-3 LCPUFA intervention and the control group, effect of the intervention, *P#* < 0.05 significant difference between male and female placentas, effect of offspring sex; *P*#* < 0.05 significant interaction between sex and the intervention. [1]last measured value at booking minus self-reported weight before pregnancy, [2]n-6/n-3 LCPUFA ratio: (C20:2n-6 + C20:3n-6 + C20:4n-6 +C22:2n-6 + C22:4n-6 +C22:5n-6) / (C20:3n-3 + C20:4n-3 + C20:5n-3 + C22:3n-3 + C22:5n-3 + C22:6n-3); Mat. RBC, maternal red blood cells; P15, gestational week-15; P32, gestational week-32; UC RBC, umbilical cord red blood cells.
