# Supplementary material for: Fetal sex modulates placental microRNA expression, potential microRNA-mRNA interactions, and levels of amino acid transporter expression and substrates: INFAT study subpopulation analysis of n-3 LCPUFA intervention during pregnancy and associations with offspring body composition
Source: BMC Mol Cell Biol. 2021 Mar 3;22:15. doi: 10.1186/s12860-021-00345-x (PMC7931339; doi:10.1186/s12860-021-00345-x)
Supplement: Supplementary file 2 — Additional file 2: Table S2. Summary of the explorative microRNA profiling of female offspring placentas. Table S3. List of expressed C19MC microRNAs identified by microRNA profiling of female offspring placentas. [file 12860_2021_345_MOESM2_ESM.pdf]

Additional file 2

Table S2 Summary of the explorative microRNA profiling of female offspring placentas

MicroRNA ID	Norm. Cq Con	Norm. Cq N3	Median Cq	FC N3 vs Con
**Upregulated microRNAs**				
hsa-miR-302b	39.63	35.22	37.42	21.22
hsa-miR-641	31.80	30.04	30.92	3.39
*hsa-miR-550**	32.37	30.70	31.53	3.17
hsa-miR-668	26.79	25.14	25.96	3.14
hsa-miR-100	15.83	15.20	15.52	1.55
hsa-miR-99a	17.81	17.20	17.51	1.53
hsa-miR-139-5p	19.05	18.45	18.75	1.51
*hsa-miR-30e**	16.49	15.96	16.22	1.45
hsa-miR-517b	17.33	16.83	17.08	1.41
hsa-miR-495	17.99	17.56	17.77	1.35
MammU6	13.79	13.40	13.59	1.31
hsa-miR-30d	16.78	16.41	16.60	1.29
**Downregulated microRNAs**				
hsa-miR-888	32.21	35.73	33.97	-11.45
hsa-miR-216a	29.77	32.69	31.23	-7.52
hsa-miR-375	23.70	26.46	25.08	-6.75
hsa-miR-581	31.38	33.56	32.47	-4.53
hsa-miR-586	30.90	33.07	31.98	-4.50
hsa-miR-923	19.86	21.34	20.60	-2.79
hsa-miR-130b	20.95	22.04	21.50	-2.12
hsa-miR-10b	20.83	21.87	21.35	-2.06
hsa-miR-223	14.51	15.05	14.78	-1.46
hsa-miR-320	16.65	17.13	16.89	-1.40
hsa-miR-21	14,93	15,38	15,15	-1,36
hsa-miR-522	15.36	15.76	15.56	-1.32
hsa-miR-451	14.96	15.34	15.15	-1.30

MicroRNA ID	Norm. Cq Con	Norm. Cq N3	Median Cq	FC N3 vs Con
**MicroRNAs detected in N3 only [Con (-) / N3 (+)]**				
hsa-miR-485-5p	BD	27.64	NA	NA
hsa-miR-361-3p	BD	29.00	NA	NA
*hsa-miR-30c-2**	BD	29.76	NA	NA
hsa-miR-219-5p	BD	32.02	NA	NA
has-miR-155	BD	32.11	NA	NA
*hsa-miR-30c-1**	BD	32.21	NA	NA
hsa-miR-208	BD	32.26	NA	NA
hsa-miR-630	BD	32.46	NA	NA
hsa-miR-219-1-3p	BD	32.92	NA	NA
*hsa-miR-497**	BD	33.05	NA	NA
*hsa-miR-130a**	BD	33.22	NA	NA
hsa-miR-936	BD	33.45	NA	NA
hsa-miR-591	BD	33.68	NA	NA
hsa-miR-767-3p	BD	33.81	NA	NA
hsa-miR-216b	BD	34.22	NA	NA
hsa-miR-569	BD	35.63	NA	N
**MicroRNAs detected in Con only [Con (+) / N3 (-)]**				
*hsa-miR-200a**	32.02	BD	NA	NA
hsa-miR-649	33.57	BD	NA	NA
hsa-miR-367	33.99	BD	NA	NA
*hsa-miR-302c**	34.84	BD	NA	NA
hsa-miR-302a	36.37	BD	NA	NA

MicroRNA profiling data (*TaqMan Low Density Human MicroRNA Assays)* of female placentas from the n-3 LCPUFA intervention group (N3) compared to the control group (Con). One pool represents 3 female placentas per group. One pool per group was analyzed. The normalized Cq (norm. Cq) after *loess* normalization are shown for each pool (Con-F and N3-F). Median Cq was calculated from normalized Cq-values. Log RQ was calculated by (norm. Cq N3-F – norm. Cq Con-F). Fold changes (FC) were calculated by 2^{logRQ} or -2^{logRQ} (in case of negative logRQ). The high and low thresholds were calculated with quantile regression with a quadratic model. LogRQ values below the 5th and above the 95th percentile were considered to be regulated and are shown in this table. Furthermore, microRNAs expressed in one group (+) and not expressed in the other group (-) are also shown and depicted as Con (-) / N3 (+) and Con (-) / N (3+). BD, Cq-value below detection limit; NA, not applicable, i.e. no median Cq or fold changes (FC) could be calculated.

Table S3 List of expressed *C19MC* microRNAs identified by microRNA profiling of female offspring placentas

Plate	Detector	Flag Con-F	Flag N3-F	Raw Cq		Norm. Cq		Median Cq	Log RQ	Threshold		Ex-treme	FC
				Con-F	N3-F	Con-F	N3-F			high	low		
B	*hsa-miR-498-4373223*	Yes	Yes	BD	BD	BD	BD	NA	NA	NA	NA	NA	NA
A	*hsa-miR-512-3p-*	No	No	14.9	12.9	13.1	13.1	13.1	0.01	0.22	-0.30	No	1.01
A	*hsa-miR-512-5p-*	No	No	18.8	18.2	18.5	18.5	18.5	-	0.42	-0.54	No	-1.03
A	*hsa-miR-515-3p-*	No	No	17.0	16.5	16.7	16.7	16.7	-	0.34	-0.45	No	-1.07
A	*hsa-miR-515-5p-*	No	No	16.6	15.6	15.6	15.9	15.8	-	0.30	-0.40	No	-1.20
B	*hsa-miR-516a-3p-*	No	No	23.9	23.5	23.7	23.7	23.7	-	0.77	-0.95	No	-1.03
A	*hsa-miR-516a-5p-*	No	No	22.4	22.2	22.6	22.4	22.5	0.21	0.67	-0.84	No	1.15
A	*hsa-miR-516b-4395172*	No	No	16.0	15.3	15.4	15.5	15.5	-	0.29	-0.39	No	-1.10
B	*hsa-miR-517*-4378078*	No	No	24.5	23.7	24.2	23.9	24.1	0.31	0.80	-0.99	No	1.24
A	*hsa-miR-517a-4395513*	No	No	14.5	11.6	11.8	11.7	11.8	0.08	0.19	-0.27	No	1.06
**A**	***hsa-miR-517b-4373244***	**No**	**No**	**18.6**	**16.5**	**17.3**	**16.8**	**17.1**	**0.50**	**0.36**	**-0.46**	**Yes**	**1.41**
A	*hsa-miR-517c-4373264*	No	No	14.7	12.0	12.1	12.2	12.1	-	0.20	-0.28	No	-1.05
A	*hsa-miR-518a-3p-*	No	No	17.9	17.1	17.7	17.4	17.6	0.30	0.38	-0.49	No	1.23
A	*hsa-miR-518a-5p-*	No	No	25.2	25.5	25.3	25.8	25.6	-	0.94	-1.15	No	-1.41
A	*hsa-miR-518b-4373246*	No	No	16.4	15.5	15.9	15.8	15.8	0.07	0.30	-0.41	No	1.05
B	*hsa-miR-518c*-*	No	No	21.3	21.1	21.0	21.3	21.2	-	0.58	-0.73	No	-1.25
A	*hsa-miR-518c-4395512*	No	No	19.3	19.0	19.1	19.2	19.2	-	0.46	-0.58	No	-1.14
A	*hsa-miR-518d-3p-*	No	No	24.3	24.5	24.5	24.8	24.6	-	0.85	-1.05	No	-1.21
B	*hsa-miR-518e*-*	No	No	22.7	22.3	22.5	22.5	22.5	-	0.67	-0.84	No	-1.04
A	*hsa-miR-518e-4395506*	No	No	15.4	14.5	14.8	14.7	14.8	0.07	0.27	-0.36	No	1.05
B	*hsa-miR-518f*-*	No	No	26.4	26.3	26.3	26.5	26.4	-	1.02	-1.25	No	-1.15
A	*hsa-miR-518f-4395499*	No	No	15.7	14.8	15.0	15.0	15.0	-	0.28	-0.37	No	-1.01
B	*hsa-miR-519b-3p-*	No	No	17.6	17.1	17.1	17.4	17.3	-	0.36	-0.47	No	-1.25
A	*hsa-miR-519c-3p-*	No	No	20.8	20.9	20.6	21.2	20.9	-	0.56	-0.71	No	-1.48
A	*hsa-miR-519d-4395514*	No	No	15.0	13.5	13.9	13.7	13.8	0.23	0.24	-0.33	No	1.18
B	*hsa-miR-519e*-*	No	No	20.6	20.2	20.4	20.4	20.4	-	0.53	-0.67	No	-1.04
A	*hsa-miR-519e-4395481*	No	No	20.7	20.2	20.7	20.5	20.6	0.25	0.54	-0.68	No	1.19
A	*hsa-miR-520a-3p-*	No	No	19.2	18.7	19.1	19.0	19.1	0.09	0.45	-0.58	No	1.06
A	*hsa-miR-520a-5p-*	No	No	20.1	19.9	20.1	20.1	20.1	0.00	0.51	-0.65	No	1.00
A	*hsa-miR-520b-4373252*	No	No	24.1	24.7	24.8	25.0	24.9	-	0.87	-1.08	No	-1.11
B	*hsa-miR-520c-3p-*	No	No	17.3	16.9	16.7	17.1	16.9	-	0.35	-0.46	No	-1.30
	hsa-miR-520d-3p	No primer present on the low-density array											
A	*hsa-miR-520e-4373255*	No	No	29.6	29.8	30.6	30.0	30.3	0.58	1.49	-1.80	No	1.50
A	*hsa-miR-520f-4373256*	No	No	23.1	22.9	23.2	23.1	23.2	0.12	0.73	-0.90	No	1.08
A	*hsa-miR-520g-4373257*	No	No	18.1	17.5	17.9	17.8	17.8	0.05	0.39	-0.51	No	1.03
B	*hsa-miR-520h-4373258*	No	No	18.9	18.3	18.5	18.5	18.5	0.03	0.42	-0.54	No	1.02
A	*hsa-miR-521-4373259*	No	No	19.1	18.9	19.0	19.2	19.1	-	0.45	-0.58	No	-1.12
**A**	***hsa-miR-522-4395524***	**No**	**No**	**16.2**	**15.5**	**15.4**	**15.8**	**15.6**	**-**	**0.29**	**-0.39**	**yes**	**-1.32**
A	*hsa-miR-523-4395497*	No	No	18.4	17.9	18.3	18.2	18.2	0.07	0.41	-0.53	No	1.05
B	*hsa-miR-524-3p-*	No	No	20.0	19.4	19.7	19.7	19.7	0.06	0.49	-0.62	No	1.04
B	*hsa-miR-524-3p-*	No	No	20.0	19.5	19.7	19.6	19.7	0.12	0.49	-0.62	No	1.09
A	*hsa-miR-525-3p-*	No	No	16.4	15.4	15.7	15.7	15.7	0.05	0.30	-0.40	No	1.03
A	*hsa-miR-525-5p-*	No	No	19.2	18.9	19.1	19.2	19.2	-	0.46	-0.58	No	-1.10
B	*hsa-miR-526b*-*	No	No	19.8	19.1	19.5	19.3	19.4	0.25	0.47	-0.60	No	1.19
A	*hsa-miR-526b-4395493*	No	No	18.5	18.0	18.4	18.3	18.4	0.03	0.41	-0.53	No	1.02
	Hsa-miR-1283	No primer present on the low-density array											
	Hsa-miR-1323	No primer present on the low-density array											

MicroRNA profiling data (*TaqMan Low Density Human MicroRNA Assays)* of female placentas from the n-3 LCPUFA intervention group (N3) compared to the control group (Con). One pool represents 3 female placentas per group. One pool per group was analyzed. The microRNA profiling was conducted on two plates, depicted as plate A or B. The columns Flag Con-F and Flag N3-F indicate problems in the amplification of the RT-qPCR. No = no problem, Yes = flagged, problem in the amplification (often flagged when there is no amplification). The raw Cq-values and the normalized Cq (norm. Cq) after *loess* normalization are shown. Median Cq was calculated from normalized Cq-values. Log RQ was calculated by (norm. Cq N3-F – norm. Cq Con-F). The high and low thresholds were calculated with quantile regression with a quadratic model. LogRQ values below the 5th and above the 95th percentile are marked with Yes in the column *Extreme*. LogRQ values within the 5th - 95th percentile are marked with No in the column *Extreme*. LogRQ values below the 5th and 95th percentile are considered to be putatively regulated. Fold changes (FC) were calculated by 2^{logRQ} or -2^{logRQ} (in case of negative logRQ). BD, below detection limit; *C19MC*, chromosome 19 microRNA cluster; NA, not applicable; §, duplicate.
