# Supplementary material for: Fetal sex modulates placental microRNA expression, potential microRNA-mRNA interactions, and levels of amino acid transporter expression and substrates: INFAT study subpopulation analysis of n-3 LCPUFA intervention during pregnancy and associations with offspring body composition
Source: BMC Mol Cell Biol. 2021 Mar 3;22:15. doi: 10.1186/s12860-021-00345-x (PMC7931339; doi:10.1186/s12860-021-00345-x)
Supplement: Supplementary file 3 — Additional file 3: Figure S1. qPCR validation data of selected placental target microRNAs. Scatter plots representing data shown in Table 3. Figure S2. qPCR validation data of selected placental target mRNAs. Scatter plots representing data shown in Table 3. Figure S3. Western blot composite of placental LAT1 and GAPDH expression and respective images of the original nitrocellulose membrane after successive indirect immunostainings and detections. [file 12860_2021_345_MOESM3_ESM.pdf]

Additional file 3: Figure S1

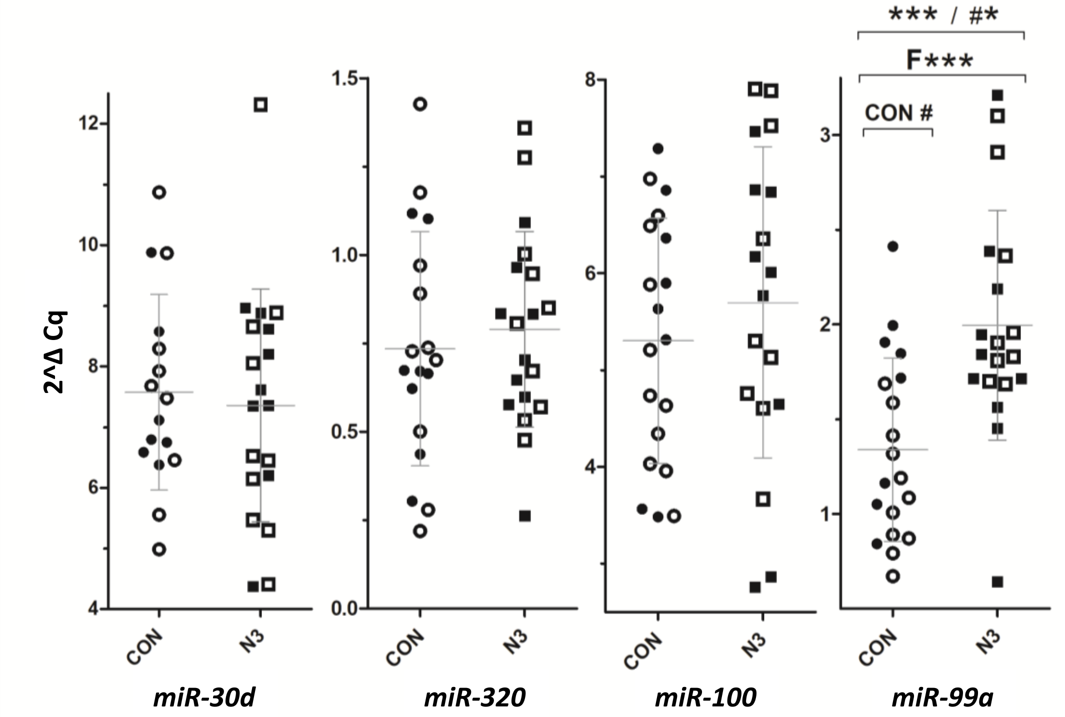

Figure S1 qPCR validation data of selected placental microRNAs. Data on microRNA expression levels (2^Δ Cq values) presented in Table 3 are shown as scatter plots with median and standard deviation. Values of placentas from the control group (CON) and the intervention group (N3) are presented with the symbols circle (CON) and square (N3), respectively. Closed black symbols represent values from male offspring placentas (circles = CON-M; squares = N3-M) and open symbols represent values from female offspring placentas (circles = CON-F; squares = N3-F). The P-values for *Treatmen*t and *Sex* are derived from linear regression models fit to the expression data without a *Sex-Group Interaction* term. The P-values for the Interaction and pairwise group comparisons (Con-M vs Con-F, N3-M vs N3-F, N3-F vs Con-F, N3M vs CM) are from regression models including a *Sex-Group Interaction* term. P-values $p < 0.05$ are considered as significant. Significant effects for the factor *Sex* are marked with #, significant effects for the factor *Treatment* are marked with *, and significant *Sex-Group Interactions* are marked with #*. * or #, two sided P-value < 0.05; ** or ##, two sided P-value < 0.01; *** or ###, two sided p-value < 0.001. Significant values only in a distinct group are marked with prefix, CON, N3, F (female placentas), and M (male placentas).

Additional file 3: Figure S2

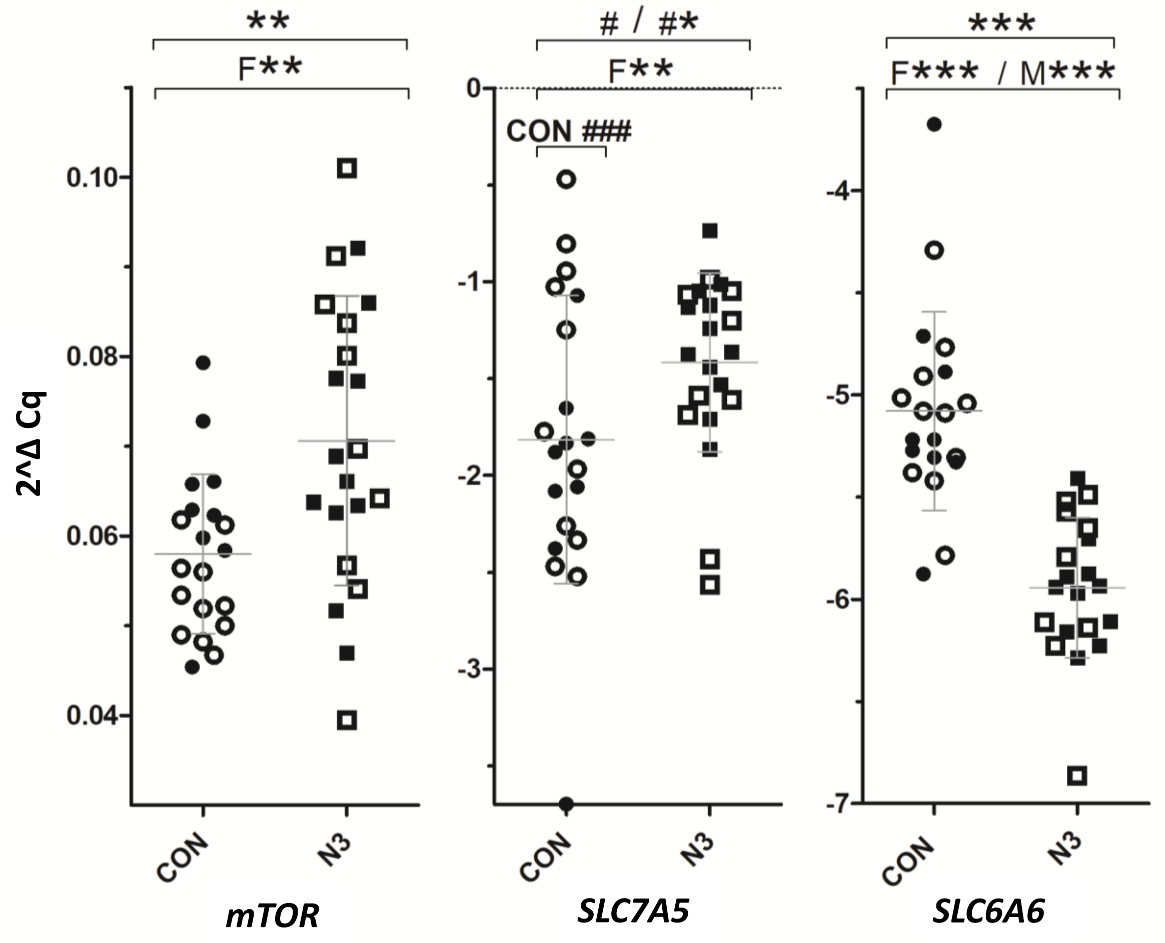

Figure S2 qPCR validation data of selected placental mRNAs. Data on mRNA expression levels (2^Δ Cq values) presented in Table 3 are shown as scatter plots with median and standard deviation. Values of placentas from the control group (CON) and the intervention group (N3) are presented with the symbols circle (CON) and square (N3), respectively. Closed black symbols represent values from male offspring placentas (circles = CON-M; squares = N3-M) and open symbols represent values from female offspring placentas (circles = CON-F; squares = N3-F). The P-values for *Treatmen*t and *Sex* are derived from linear regression models fit to the expression data without a *Sex-Group Interaction* term. The P-values for the Interaction and pairwise group comparisons (Con-M vs Con-F, N3-M vs N3-F, N3-F vs Con-F, N3M vs CM) are from regression models including a *Sex-Group Interaction* term. P-values $p < 0.05$ are considered as significant. Significant effects for the factor *Sex* are marked with #, significant effects for the factor *Treatment* are marked with *, and significant *Sex-Group Interactions* are marked with #*. * or #, two sided P-value < 0.05; ** or ##, two sided P-value < 0.01; *** or ###, two sided p-value < 0.001. Significant values only in a distinct group are marked with prefix, CON, N3, F (female placentas), and M (male placentas).

Additional file 3: Figure S3

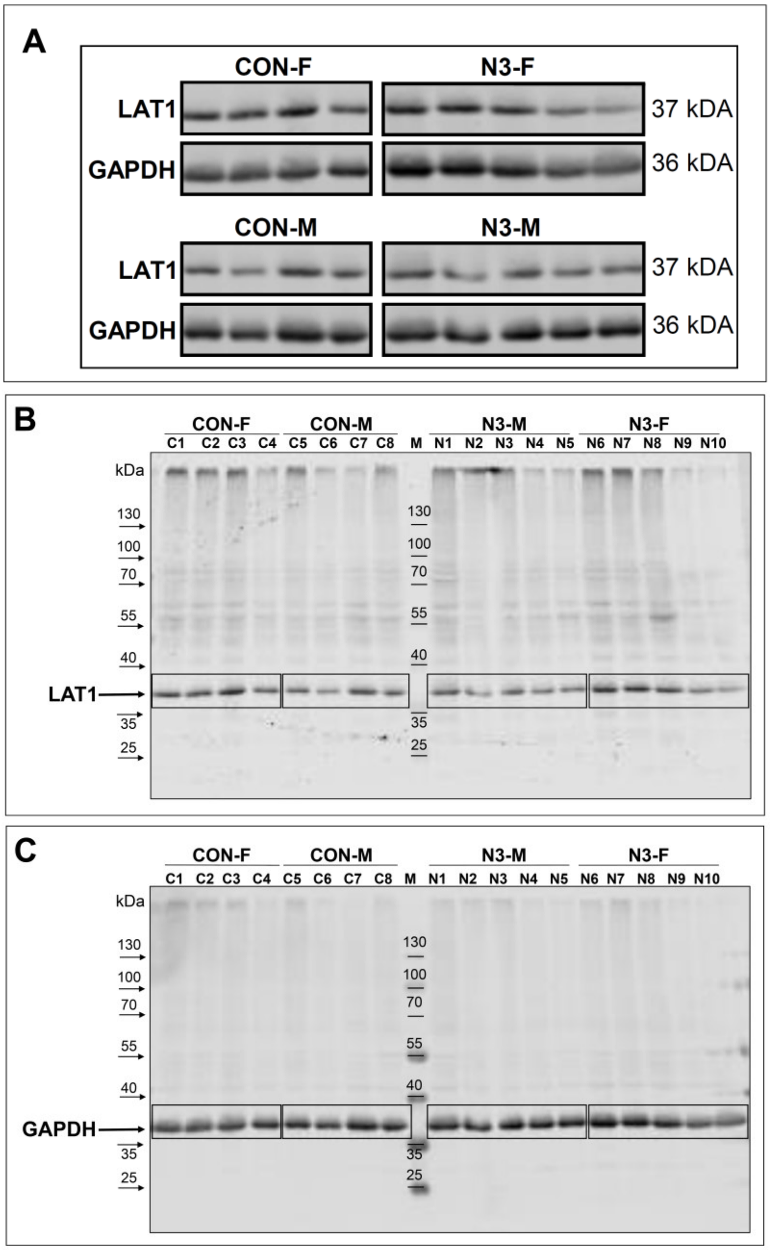

Figure S3: **Western blot composite of placental LAT1 and GAPDH expression and respective images of the original nitrocellulose membrane after successive indirect immunostainings and detections.**

A – C Image of western blot composite for placental LAT1 and GAPDH expression (**A**; see also Figure 3) and respective images of the original nitrocellulose membrane after successive indirect immunostainings and detections of LAT1 (**B**) and GAPDH (**C**) are shown (grey scale black on white of respective channels). Specific single panel areas of the composite (**A**) taken from the images of the membrane are depicted in corresponding black frames in **B** (800 nm channel) and **C** (700 nm channel). Prestained molecular weight ladder for SDS-PAGE was loaded in lane **M.** Prestained molecular weight ladder proteins can also be visualized on the membrane via near-infrared fluorescence lasers, especially in the 700 nm channel as used for detection of GAPDH. Positions of the specific proteins (LAT1, GAPDH) are labelled and marked with arrows. Molecular weights (kDa) and positions of the protein ladder are labbeled and marked with arrows (left margin) and solid short lines (lane M), respectively.

Abbreviations: **Con-F**, female offspring of the control group; **C1** – **C4**, protein extracts of Con-F. **Con-M**, male offspring of the control group; **C5** – **C8**, protein extracts of Con-M. **N3-F**, female offspring of the n-3 LCPUFA intervention group; **N6** – **N10** protein extracts of N3-F. **N3-M**, male offspring of the n-3 LCPUFA intervention group; **N1** – **N5** protein extracts of N3-F. **M**, prestained molecular weight ladder for SDS-PAGE (PageRuler, Fermentas) with respective protein weights in kDa**.**

Methods: In brief, placental protein samples (50 µg) of respective female (C1 – C4; N6 – N10) and male (C5 – C8;

Legend continued on next page

Legend to Figure S3 continued

N1 – N5) offspring from the control (CON) and intervention group (N3) were first separated by SDS-PAGE using a 10% SDS-polyacrylamide gel. Afterwards, protein transfer onto a nitrocellulose membrane was performed. Subsequently, successive indirect immunostainings for the target protein LAT1 (37 kDa) and for the housekeeper protein GAPDH (36 kD) were applied. *LI-COR ODYSSEY* infrared imaging system and software were used for detection and protein band intensity quantification (LAT1, channel 800 nm; GAPDH, channel 700), respectively. GAPDH was used as housekeeper protein for expression normalization (for further details see Methods part).
