# Supplementary material for: Fetal sex modulates placental microRNA expression, potential microRNA-mRNA interactions, and levels of amino acid transporter expression and substrates: INFAT study subpopulation analysis of n-3 LCPUFA intervention during pregnancy and associations with offspring body composition
Source: BMC Mol Cell Biol. 2021 Mar 3;22:15. doi: 10.1186/s12860-021-00345-x (PMC7931339; doi:10.1186/s12860-021-00345-x)
Supplement: Supplementary file 4 — Additional file 4: Table S4. Summary of data from Sedlmeier et al. [10] on placental free estradiol-17ß (E2) and testosterone (T) levels, and E2/T ratios. Table S5. Associations of maternal plasma taurine and tryptophan at week-32 of gestation with placental expression and fetal taurine and tryptophan. Table S6. Associations of regulated placental gene and protein expression levels with taurine and tryptophan levels in fetal compartments. Table S7A. Model 1: unadjusted associations of regulated placental gene and protein expression levels with offspring body composition. Table S7B. Model 2: adjusted associations of regulated placental gene and protein expression levels with offspring body composition. Table S8A. Model 1: unadjusted associations of levels of taurine and tryptophan in placental tissue and cord and maternal plasma with offspring body composition. Table S8B. Model 2: adjusted associations of levels of taurine and tryptophan in placental tissue and cord and maternal plasma with offspring body composition. [file 12860_2021_345_MOESM4_ESM.pdf]

Additional file 4

Table S4: Summary of data from Sedlmeier et al [10] on placental free estradiol-17ß (E2) and testosterone (T) levels, and E2/T ratios

Sex steroid hormones		Male			Female				*P**	*P#*	*P*#*
		N	Median	(25th-75th percentile)	N	Median	(25th-75th percentile)	**Con-M vs. Con-F**	**N3-M vs. N3-F**	**N3-F vs. Con-F**	**N3-M vs. Con-M**
Placental free estradiol-17ß (ng/g)†	**Con**	9	53.2	(40.1-86.1)	11	50.9	(42.7-70.3)		0.830	0.396	0.616
	**N3**	11	65.4	(35.4-82.4)	10	50.6	(25.5-74.8)				
Placental testosterone (ng/g)†	**Con**	9	10.6	(7.9-16.6)	11	14.8	(10.2-20.1)		0.252	**0.008**	0.278
	**N3**	11	10.7	(9.7-14.4)	10	19.7	(16.6-22.6)	0.249	**0.008**	0.113	0.965
Placental free estradiol-17ß / testosterone ratio†	**Con**	9	5.0	(3.0-6.6)	11	4.2	(2.5-6.1)		0.398	**0.013**	**0.045**
	**N3**	11	5.7	(3.6-6.3)	10	2.1	(1.7-3.7)	0.719	**0.002**	**0.042**	0.401

Sex steroid hormone data are presented as median with interquartile range (IQR: 25th - 75th percentiles). Statistical significance was calculated by two-way ANOVA with Holm-Sidak *post-hoc* test. †Not normal distributed sex steroid hormone parameters were tested for statistical significance by two-way ANOVA on ranks with Holm-Sidak *post-hoc* test. *P*, P-value; #, significant effects for sex differences; *, significant effects for the n-3 LCPUFA treatment; * #, significant interactions. *P* < 0.05 are marked in bold. Con, control group; N3, intervention group; Con-M; placentas of male offspring in the control group; Con-F; placentas of female offspring in the control group; N3-M, placentas of male offspring in the n-3 LCPUFA intervention group, N3-F, placentas of female offspring in the n-3 LCPUFA intervention group.

Table S5 Associations of maternal plasma taurine and tryptophan at week-32 of gestation with placental expression and fetal taurine and tryptophan

Outcome variables	N	P32 Taurine beta (95% CI)	*P*	P32 Tryptophan beta (95% CI)	*P*
**Model 1**					
mTOR	39	0 (−0.01; 0.01)	0.989	0.01 (−0.01; 0.03)	0.223
miR-99a	37	0 (−0.01; 0.01)	0.920	0.02 (−0.02; .05)	0.332
SLC6A6	38	0.01 (0; 0.02)	0.178	−0.02 (−0.06; 0.02)	0.258
SLC7A5	39	0 (−0.01; 0.01)	0.738	0.02 (−0.02; 0.06)	0.314
LAT1	16	0 (0; 0)	0.215	0 (−0.01; 0)	0.883
PL Taurine	38	1.88 (−1.63; 5.38)	0.285	−4.21 (−18.29; 9.88)	0.548
UC Taurine	32	−0.84 (−5.74; 4.05)	0.727	−10.78 (−28.03; 6.47)	0.212
PL Tryptophan	38	0.01 (−0.01; 0.03)	0.458	−0.01 (−0.08; 0.07)	0.849
UC Tryptophan	32	−0.42 (−1.12; 28)	0.227	**3.38 (1.11; 5.64)**	**0.005**
**Model 2**					
mTOR	39	0 (0; 0.01)	0.303	0.01(0.01; 0.04)	0.370
miR-99a	37	0.01 (0; 0.02)	0.234	−0.01 (−0.06; 0.04)	0.647
SLC6A6	38	0 (−0.01; 0.01)	0.611	0 (−0.03; 0.04)	0.861
SLC7A5	39	0 (−0.01; 0.01)	0.450	0 (−0.05; 0.05)	0.909
LAT1	16	0 (0; 0)	0.200	0 (−0.01; 0.01)	0.512
PL Taurine	38	1.65 (−2.73; 6.03)	0.449	1.80 (−18.52; 22.11)	0.858
UC Taurine	32	0.05 (−5.40; 5.50)	0.985	−15.92 (−41.91; 10.07)	0.220
PL Tryptophan	38	0.01 (−0.02; 0.03)	0.564	0.05 (−0.05; 0.16)	0.311
UC Tryptophan	32	−0.34 (−1.13; 0.45)	0.385	**3.83 (0.33; 7.33)**	**0.033**

Linear regression analyses: complete data set of associations of maternal plasma taurine and tryptophan at week-32 of gestation with placental *mTOR mRNA*, *miR-99a*, SLC6A6 mRNA, *SLC7A5* mRNA and LAT1 with taurine (Tau) and tryptophan (Trp) is shown. ΔCq-values for mRNA and microRNA expression of respective genes were used; optical density values for LAT1 protein expression normalized to respective GAPDH protein from western blot experiments were used. Amino acid levels (µmol/L)-values for maternal plasma levels at week-32 of gestation (P32) and umbilical cord plasma (UC) were used; amino acid levels (µmol/g protein)-values for placental tissue (PL) were used; Beta (95% CI) values with *P*-values < 0.05 are given in bold. Abbreviations: N, sample numbers analyzed; *P*, *P*-value.

Model 1: unadjusted

Model 2: adjusted for respective baseline levels of maternal Tau and Trp levels at week-15 of gestation (P15) before treatment, sex, and group

Table S6 Associations of regulated placental gene and protein expression levels with taurine and tryptophan levels in fetal compartments

Outcome variables	N	*mTOR* beta (95% CI)	*P*	N	*miR-99a* beta (95% CI)	*P*	N	*SLC6A6* beta (95% CI)	*P*	N	*SLC7A5* beta (95% CI)	*P*	N	LAT1 beta (95% CI)	*P*
**Model 1**															
PL Taurine	**40**	**255.92(32.50; 479.34)**	**0.026**	37	88.04 (−48.01; 224.09)	0.197	39	28.14 (−70.95; 127.23)	0.568	40	48.91 (−68.21; 166.03)	0.403	17	26.52 (−91.42; 144.45)	0.639
UC Taurine	34	−192.24 (−478.48; 94.00)	0.181	32	−44.73 (−214.75; 125.28)	0.595	33	36.93 (−113.39; 187.24)	0.620	34	65.27 (−82.22; 212.27)	0.374	16	194.53 (−17.01; 406.07)	0.069
PL Tryptophan	40	1.10 (−0.14; 2.35)	0.080	37	0.45 (−0.29; 1.19)	0.222	39	0.06 (−0.55; 0.67)	0.842	40	0.23 (−0.41; 0.87)	0.467	17	0.13 (−0.76; 1.02)	0.760
UC Tryptophan	34	26.65 (−14.84; 63.13)	0.200	32	4.25 (−20.62; 29.13)	0.729	33	−15.85 (−37.15; 5.46)	0.139	34	−11.12 (−32.34; 10.10)	0.294	16	3.37 (−25.76; 32.50)	0.808
**Model 2**															
PL Taurine	**40**	**325.06 (77.33; 572.78)**	**0.012**	37	110.27 (−55.54; 276.08)	0.185	39	10.41 (−132.86; 153.68)	0.884	40	73.16 (−67.79; 214.11)	0.299	17	56.15 (−146.28; 258.58)	0.557
UC Taurine	34	−282.65 (−616.79; −51.49)	0.094	32	−81.11 (−288.20; 125.95)	0.429	33	134.71 (−93.90; 363.32)	0.238	34	83.33 (−91.10; 265.75)	0.325	**16**	**476.02 (−139.63; 812.42)**	**0.010**
PL Tryptophan	**40**	**1.46 (0.13; 2.80)**	**0.033**	37	0.66 (−0.19; 1.52)	0.122	39	−0.07 (−0.96; 0.82)	0.868	40	0.54 (−0.16; 1.24)	0.125	17	0.03 (−1.39; 1.44)	0.968
UC Tryptophan	34	27.86 (−20.53; 76.24)	0.249	32	−6.43 (−35.94; 23.09)	0.659	33	−17.74 (−51.19; 15.70)	0.286	34	−17.50 (−40.79; 5.80)	0.135	16	−30.45 (−75.29; 14.39)	0.163

Linear regression analyses: complete data set of associations of placental *mTOR* mRNA, *miR-99a, SLC6A6* mRNA, and *SLC7A5* mRNA, and LAT1 with taurine (Tau) and tryptophan (Trp) in placenta (PL) and umbilical cord plasma (UC) is shown. ΔCq-values for mRNA and microRNA expression of respective genes were used; optical density values for LAT1 protein expression normalized to respective GAPDH protein from western blot experiments were used. Amino acid levels (µmol/g protein)-values were used for placental tissue (PL); plasma amino acid levels (µmol/L)-values were used for umbilical cord plasma (UC). Beta (95% CI) values with *P*-values < 0.05 are given in bold. Abbreviations: N, sample numbers analyzed; *P*, *P*-value.

Model 1: unadjusted

Model 2: adjusted for respective baseline levels of maternal Tau and Trp levels at week-15 of gestation (P15) before treatment, sex, and group

Table S7A Model 1: unadjusted associations of regulated placental gene and protein expression levels with offspring body composition

Body composition variables	N	*mTOR* beta (95% CI)	*P*	N	*miR-99a* beta (95% CI)	*P*	N	*SLC6A6* beta (95% CI)	*P*	N	*SLC7A5* beta (95% CI)	*P*	N	LAT1 beta (95% CI)	*P*
**Birth**															
Placental weight (g)	41	−67.32 (−145.04; 10.41)	0.088	38	10.58 (−35.86; 57.03)	0.647	40	−2.28 (45.61; 41.05)	0.916	41	−33.86 (−72.20; 4.47)	0.082	18	54.64 (−28.22; 137.50)	0.181
BW/PW ratio	**41**	**1.04 (0.10; 1.99)**	**0.032**	38	−0.44 (−0.99; 0.12)	0.118	40	0 (−0.54; 0.54)	0.996	41	0.37 (−0.12; 0.85)	0.132	18	−0.47 (−1.53; 0.59)	0.357
Birthweight (g)	41	143.51 (−126.16; 413.19)	0.288	38	−122.37 (−272.82; 38.08)	0.108	40	20.34 (−124.53; 165.22)	0.778	41	−22.06 (−157.04; 112.91)	0.743	18	155.27 (−82.58; 393.12)	0.185
Weight/Length ratio	41	1.42 (−3.56; 6.41)	0.567	38	−2.65 (−5.36; 0.07)	0.056	40	0.18 (−2.50; 2.86)	0.894	41	−0.95 (−3.41; 1.50)	0.436	18	2.78 (−2.15; 7.70)	0.250
Fat mass (g)	39	55.21 (−47.34; 157.75)	0.282	36	−15.20 (77.53; 47.13)	0.623	38	−4.97 (−61.83; 51.88)	0.860	39	−6.61 (−44.48; 57.71)	0.795	18	77.69 (−11.25; 166.63)	0.083
Lean mass (g)	39	86.93 (−133.42; 307.29)	0.429	36	−95.99 (−225.10; 33.13)	0.140	38	12.84 (−106.68; 132.36)	0.829	39	−26.47 (−135.22; 82.27)	0.625	18	77.58 (−126.09; 281.24)	0.431
**1 year**															
Weight (g)	40	636.18 (−484.06; 1756.43)	0.257	37	155.67 (−411.24; 722.58)	0.581	39	−120.25 (−715.37; 474.86)	0.685	**40**	**558.89 (44.09; 1073.68)**	**0.034**	17	−440.172(−1625.46; 745.11)	0.441
Fat mass (g)	40	221.80 (−183.63; 627.23)	0.275	37	95.41 (−121.43; 312.24)	0.378	39	−5.57 (−220.57; 209.43)	0.958	40	168.56 (−21.09; 358.20)	0.080	17	−94.8.63 (−526.88; 337.16)	0.646
Lean mass (g)	40	414.39 (−385.74; 1214.52)	0.301	37	60.26 (−340.53; 461.05)	0.762	39	−114.68(−538.12; 308.75)	0.586	**40**	**390.32 (22.79; 757.87)**	**0.038**	17	−345.31 (−1153.98; 463.36)	0.377
**3 years**															
Weight (g)	36	563.43 (−1582.88; 2709.75)	0.597	33	96.97 (−852.35; 1046.30)	0.836	35	−601.84 (−2031.26; 827.58)	0.398	36	971.58 (−47.11; 1990.26)	0.061	15	−266.40 (−2822.27;2289.48)	0.825
Fat mass (g)	22	483.04 (−604.20; 1570.28)	0.365	20	244.06 (−199.38; 687.50)	0.263	21	−349.84 (−1050.81; 351.13)	0.309	**22**	**492.81 (10.88; 974.73)**	**0.045**	10	−714.4.30 (−2350.34; 921.48)	0.343
Lean mass (g)	22	319.95 (−1979.42; 2619.32)	0.775	20	−142.18 (−852.56; 568.19)	0.679	21	−23.26 (−1511.75; 1465.23)	0.974	22	541.14 (−537.77; 1620.05)	0.308	10	−899.24 (−4408.57;2610.10)	0.571
**5 years**															
Weight (g)	34	1246.40 (−2350.44; 4843.25)	0.485	31	223.28 (−1077.11; 1523.68)	0.728	33	−760.56 (−3033.74; 1512.61)	0.500	34	1318.28 (−256.63; 2893.18)	0.098	14	−352.18 (−4541.94; 38374)	0.858
Fat mass (g)	23	469.91 (−1088.47; 2028.29)	0.537	21	318.68 (−340.91; 978.26)	0.325	22	−394.24 (−1457.60; 669.13)	0.448	23	539.54 (−169.71; 1248.79)	0.129	11	−653.10 (−2979.92; 1673.73)	0.541
Lean mass (g)	23	1038.95 (−2426.56; 4504.46)	0.540	21	24.31 (−1099.66; 1148.27)	0.964	22	−228.34 (−2628.35; 2171.67)	0.845	23	895.84 (−722.26; 2513.94)	0.263	11	−1308.88 (−6459.88; 3842.12)	0.579

Unadjusted linear regression analyses (Model 1): complete data set of associations is shown. ΔCq-values for mRNA and microRNA expression of respective genes were used; optical density values for LAT1 protein expression normalized to respective GAPDH protein from western blot experiments were used. Beta (95% CI) values with *P*-values < 0.05 are given in bold. Abbreviations: BW, birthweight; N, sample numbers analyzed; *P*, *P*-value; PW, placental weight.

Table S7B Model 2: adjusted associations of regulated placental gene and protein expression levels with offspring body composition

Body composition variables	N	*mTOR*		N	*miR-99a*		N	*SLC6A6*		N	*SLC7A5*		N	LAT1	
		beta (95% CI)	*P*		beta (95% CI)	*P*		beta (95% CI)	*P*		beta (95% CI)	*P*		beta (95% CI)	*P*
**Birth**															
Placental weight (g)	41	−66.64 (−155.76; 22.48)	0.138	38	25.94 (−29.17; 81.05)	0.346	40	−31.95 (−95.24; 31.33)	0.313	41	−35.85 (−80.41; 8.71)	0.112	18	13.91 (−112.20; 140.03)	0.816
BW/PW ratio	41	1.00 (−0.09; 2.08)	0.071	**38**	**−0.76 (−1.40;− 0.13)**	**0.019**	40	0.40 (−0.38; 1.19)	0.307	41	0.32 (−0.24; 0.88)	0.262	18	0.27 (−1.36; 1.89)	0.732
Birthweight (g)	41	181.39 (−126.51; 489.29)	0.240	38	−144.99 (−325.89; 35.91)	0.113	40	22.56 (−194.02; 239.14)	0.834	41	−25.47 (−182.84; 131.90)	0.745	18	200.07 (−183.32; 583.46)	0.282
Weight/Length ratio	41	2.41 (−3.25; 8.08)	0.394	38	−2.84 (−6.12; 0.44)	0.088	40	−0.57 (−4.57; 3.42)	0.772	41	−0.85 (−3.71; 2.01)	0.553	18	1.97 (−5.95; 9.90)	0.602
Fat mass (g)	39	50.10 (−64.37; 164.58)	0.380	36	−25.65 (−97.65; 46.35)	0.473	38	31.93 (−49.62; 113.48)	0.432	39	13.64 (−43.53; 70.82)	0.631	18	73.26 (−72.40; 218.92)	0.299
Lean mass (g)	39	120.67 (−133.03; 374.36)	0.341	36	−104.34 (−258.65; 49.97)	0.178	38	−11.85 (−190.66; 166.96)	0.894	39	−34.68 (−161.50; 92.15)	0.582	18	126.81 (−190.87; 444.50)	0.406
**1 year**															
Weight (g)	40	230.72 (−990.98; 1452.42)	0.704	37	65.59 (−619.10; 754.29)	0.842	39	36.07 (−816.60; 888.74)	0.932	40	298.86 (−328.20; 925.92)	0.340	17	−432.04 (−2431.91; 1567.84)	0.646
Fat mass (g)	40	165.08 (−298.28; 628.43)	0.474	37	103.05 (−168.05; 374.15)	0.444	39	42.21 (−282.18; 366.60)	0.793	40	143.14 (−94.10; 380.38)	0.229	17	−288.78 (−1020.70; 443.14)	0.407
Lean mass (g)	40	65.65 (−786.82; 918.11)	0.877	37	−35.46 (−510.85; 439.93)	0.880	39	−6.14(−601.13; 588.86)	0.983	40	155.72 (−283.62; 595.07)	0.477	17	−143.26 (−1474.39; 1187.88)	0.819
**3 years**															
Weight (g)	36	−235.01 (−2566.04; 2096.02)	0.838	33	−301.60 (−1507.19; 903.99)	0.612	35	167.75 (−2011.30; 2346.80)	0.876	36	562.71 (−730.36; 1855.78)	0.382	15	360.33 (−4273.49; 4994.15)	0.866
Fat mass (g)	22	250.96 (−898.06; 1399.99)	0.651	20	195.33 (−449.97; 840.63)	0.529	21	51.18 (−1126.09; 1228.44)	0.928	22	362.20 (−207.62; 932.02)	0.198	10	−1026.58 (−3764.31; 1711.15)	0.379
Lean mass (g)	22	−127.58 (−2546.75; 2291.58)	0.913	20	−404.47 (−1468.55; 659.61)	0.430	21	304.36 (−2080.58; 2689.30)	0.790	22	127.01 (−1125.52; 1379.54)	0.833	10	−819.91 (−6353.45; 4713.64)	0.719
**5 years**															
Weight (g)	34	−163.35 (−4113.98; 3787.27)	0.933	31	−54.59 (−1136.62; 1627.45)	0.947	33	510.49 (−2809.95; 3830.93)	0.755	34	608.94 (−1346.48; 2564.37)	0.529	14	793.59 (−7088.11; 8675.29)	0.825
Fat mass (g)	23	329.67 (−1215.82; 1875.15)	0.659	21	332.82 (−412.76; 1078.40)	0.358	22	191.05 (−1307.17; 1689.27)	0.791	23	437.27 (−309.48; 1184.02)	0.234	11	−1039.324(−4817.51;2738.87)	0.526
Lean mass (g)	23	129.48 (−3416.43; 36754.40)	0.940	21	−242.50(−1667.15; 1182.14)	0.723	22	748.87 (−2627.52; 4125.27)	0.646	23	166.60 (−1605.80; 1939.00)	0.846	11	−376.96 (−9139.81; 8385.89)	0.920

Adjusted linear regression analyses (Model 2): complete data set of associations is show. ΔCq-values for mRNA and microRNA expression of respective genes were used; optical density values for LAT1 protein expression normalized to respective GAPDH protein from western blot experiments were used. Beta (95% CI) values with *P*-values < 0.05 are given in bold. Abbreviations: BW, birthweight; N, sample numbers analyzed; *P*, *P*-value; PW, placental weight.

Model 2: adjusted for sex and group. From 1 year, adjusted for breastfeeding status at 4 months (partially breastfed/formula fed or fully breastfed)

Table S8A Model 1: unadjusted associations of levels of taurine and tryptophan in placental tissue and cord and maternal plasma with offspring body composition

Body composition variables	N	PL Taurine<br>beta (95% CI)	*P*	N	UC Taurine<br>beta (95% CI)	*P*	N	P32 Taurine<br>beta (95% CI)	*P*	N	PL Tryptophan<br>beta (95% CI)	*P*	N	UC Tryptophan<br>beta (95% CI)	*P*	N	P32 Tryptophan<br>beta (95% CI)	*P*
**Birth**																		
Placental weight (g)	40	−0.09 (−0.20; 0.01)	0.083	34	0.07 (−0.04; 0.18)	0.186	39	0.19 (−1.01; 1.38)	0.753	40	−14.28 (−33.81; 5.25)	0.147	34	0.20 (−0.56; 0.95)	0.602	39	−1.31 (−6.03; 3.42)	0.579
BW/PW ratio	40	0 (0; 0)	0.109	34	0 (0; 0)	0.471	39	0 (−0.02; 0.01)	0.608	40	0.13 (−0.13; 0.38)	0.311	34	0 (−0.01; 0.01)	0.923	39	0 (−0.06; 0.06)	0.994
Birthweight (g)	40	0.04 (−0.33; 0.41)	0.830	34	0.19 (−0.17; 0.53)	0.289	39	0.35 (−3.72; 4.42)	0.862	40	−8.87 (−77.02; 59.28)	0.794	34	1.43 (−0.97; 3.84)	0.233	39	−5.33 (−21.45; 10.78)	0.507
Weight/Length ratio	40	0 (−0.01; 0.01)	0.596	34	0.01 (0; 0.01)	0.058	39	0 (0.07; 0.08)	0.957	40	−0.43 (−1.68; 0.81)	0.486	34	0.02 (−0.02; 0.07)	0.338	39	−0.18 (−0.47; 0.11)	0.218
Fat mass (g)	38	0.01 (−0.14; 0.15)	0.942	33	0.05 (−0.10; 0.20)	0.487	37	0.58 (−0.96; 2.11)	0.451	38	−6.53 (−32.59; 19.54)	0.615	33	0.28 (−0.73; 1.28)	0.579	37	2.67 (−3.49; 8.84)	0.385
Lean mass (g)	38	0.03 (−0.27; 0.34)	0.830	33	0.11 (−0.17; 0.39)	0.426	37	−0.08 (−3.39; 3.24)	0.962	38	−3.65 (−60.12; 52.83)	0.897	33	1.14 (−0.74; 3.01)	0.225	37	−9.69 (−22.65; 3.27)	0.138
**1 year**																		
Weight (g)	39	−0.35 (−1.84; 1.14)	0.633	33	0.22 (−1.08; 1.52)	0.730	**38**	**−18.34 (−33.69; 2.99)**	**0.021**	39	−37.04 (−314.12; 240.05)	0.788	33	4.74 (−3.90; 13.36)	0.272	38	48.26 (−15.59; 112.11)	0.134
Fat mass (g)	39	0.04 (−0.50; 0.59)	0.871	33	0.04 (−0.49; 0.58)	0.871	38	−3.10 (−8.96; 2.77)	0.291	39	13.72 (−87.50; 114.93)	0.785	33	0.44 (−3.15; 4.03)	0.805	38	10.58 (−12.85; 34.02)	0.366
Lean mass (g)	39	−0.40 (−1.46; 0.66)	0.452	33	0.18 (−0.72; 1.08)	0.688	**38**	**−15.24 (−25.83; −4.65)**	**0.006**	39	−50.76 (−248.53; −147.02)	0.606	33	4.30 (−1.59; 10.19)	0.147	38	37.68 (−7.45; 82.81)	0.099
**3 years**																		
Weight (g)	36	−1.09 (−3.99.; 1.82)	0.453	29	1.33 (−1.07.; 3.73)	0.265	**34**	**−30.90 (−60.30; −1.46)**	**0.040**	36	−94.97 (−670.79; 480.84)	0.740	29	8.83 (−6.74; 24.40)	0.255	34	87.12 (−36.64; 210.89)	0.161
Fat mass (g)	22	−0.21 (−1.64; 1.22)	0.763	18	−0.57 (−2.11; 0.97)	0.446	22	−8.50 (−22.44; 5.43)	0.218	22	79.26 (−234.07; 392.59)	0.604	18	7.16 (−1.61; 15.93)	0.103	22	53.15 (−8.32; 114.61)	0.086
Lean mass (g)	22	−0.30 (−3.27; 2.66)	0.834	18	−0.42 (−3.02; 2.18)	0.737	22	−20.72 (−49.20; 7.75)	0.145	22	184.46 (−464.61; 833.52)	0.560	18	**14.68 (0.83; 28.54)**	**0.039**	22	107.09 (−21.06;235.23)	0.097
**5 years**																		
Weight (g)	34	−0.27 (−4.92; 4.39)	0.908	28	1.04 (−2.42; 4.50)	0.543	32	−42.86 (−95.90; 0.20)	0.051	34	220.34 (−646.56; 1087.23)	0.608	28	20.36 (−5.80; 46.52)	0.122	32	105.16 (−96.10;306.42)	0.294
Fat mass (g)	23	0.18 (−1.79; 2.15)	0.853	20	−1.04 (−2.93; 0.85)	0.263	23	−14.78 (−34.58; 5.02)	0.136	23	131.37 (−233.88; 496.61)	0.463	20	7.33 (−5.88; 20.53)	0.259	23	51.01 (−31.25; 133.26)	0.211
Lean mass (g)	23	−0.78 (−5.14; 3.59)	0.714	20	0.55 (−2.76; 3.85)	0.732	23	−39.44 (−82.34; 3.45)	0.070	23	78.42 (−743.70; 900.53)	0.845	**20**	**23.19 (3.11; 43.27)**	**0.026**	23	74.75 (−112.21; 261.70)	0.415

Unadjusted linear regression analyses (Model 1): complete data set of associations is shown. Amino acid levels (µmol/g protein)-values for placental tissue (PL) were used; amino acid levels (µmol/L)-values for umbilical cord plasma (UC) and maternal plasma levels at week-32 of gestation (P32) were used. Beta (95% CI) values with *P*-values < 0.05 are given in bold. Abbreviations: BW, birth weight; N, sample numbers analyzed; *P*, *P*-value; PW, placental weight.

Table S8B Model 2: adjusted associations of levels of taurine and tryptophan in placental tissue and cord and maternal plasma with offspring body composition

Body composition variables	N	PL Taurine beta (95% CI)	*P*	N	UC Taurine beta (95% CI)	*P*	N	P32 Taurine beta (95% CI)	*P*	N	PL Tryptophan beta (95% CI)	*P*	N	UC Tryptophan beta (95% CI)	*P*	N	P32 Tryptophan beta (95% CI)	*P*
**Birth**																		
Placental weight (g)	40	−0.09 (−0.20; 0.01)	0.100	34	0.07 (−0.05; 0.18)	0.229	39	0.19 (−1.31; 1.68)	0.802	40	−15.64 (−36.83; 5.55)	0.143	34	0.31 (−0.52; 1.13)	0.456	39	−1.28 (−8.35; 5.79)	0.715
BW/PW ratio	40	0 (0; 0)	0.113	34	0 (0; 0)	0.511	39	0 (−0.02; 0.02)	0.684	40	0.13 (−0.15; 0.40)	0.357	34	0 (−0.01; 0.01)	0.900	39	0.01 (−0.08; 0.09)	0.877
Birthweight (g)	40	0.04 (−0.36; 0.43)	0.851	34	0.20 (−0.18; 0.57)	0.294	39	−0.01 (−5.15; 5.12)	0.996	40	−18.60 (−92.74;55.56)	0.614	34	1.94 (−0.62; 4.49)	0.132	39	0.52 (−23.32; 24.36)	0.965
Weight/Length ratio	40	0 (−0.01; 0.01)	0.593	34	0.01 (0; 0.01)	0.065	39	−0.01 (−0.10; 0.09)	0.871	40	−0.75 (−2.07; 0.58)	0.262	34	0.03 (−0.02; 0.08)	0.173	39	−0.08 (−0.51; 0.36)	0.727
Fat mass (g)	38	0.02 (−0.17; 0.13)	0.777	33	0.06 (−0.09; 0.21)	0.411	37	0.95 (−0.89; 2.79)	0.300	38	−9.28 (−37.41; 18.85)	0.507	33	0.09 (−0.99; 1.16)	0.870	37	1.24 (−8.03; 10.50)	0.788
Lean mass (g)	38	0.04 (−0.29; 0.37)	0.809	33	0.11 (−0.18; 0.41)	0.439	37	−0.71 (−4.90; 3.48)	0.731	38	−14.77 (−75.07; 45.53)	0.622	33	1.78 (−0.09; 3.65)	0.061	37	−5.24 (−25.00; 14.53)	0.593
**1 year**																		
Weight (g)	39	−0.32 (−1.82; 1.18)	0.663	33	0.45 (−0.96; 1.86)	0.518	38	−15.03 (−33.47; −3.41)	0.107	39	−134.60 (−429.80; 160.60)	0.360	33	5.90 (−3.35; 15.14)	0.202	**38**	**86.19 (−0.30; 172.08)**	**0.049**
Fat mass (g)	39	0.03 (−0.56; 0.61)	0.923	33	0.14 (−0.46; 0.74)	0.632	38	−2.08 (−9.44; 5.28)	0.570	39	−24.97 (−137.66; 87.72)	0.655	33	0.67 (−3.23; 4.57)	0.728	38	24.49 (−8.67; 57.64)	0.142
Lean mass (g)	39	−0.35 (−1.39; 0.68)	0.494	33	0.31 (−0.65; 1.27)	0.517	**38**	**−12.95 (−25.41; −0.49)**	**0.042**	39	−109.63 (−317.87; −98.62)	0.292	33	5.23 (−1.06; 11.51)	0.099	**38**	**61.70 (1.49; 121.91)**	**0.045**
**3 years**																		
Weight (g)	36	−0,95 (−3.89.; 1.98)	0.512	29	1.43 (−1.16.; 4.02)	0.265	34	−20.89 (−60.27; 18.49)	0.287	36	−285.19 (−861.32; 350.94)	0.397	29	9.18 (−7.89; 26.26)	0.277	34	158.70 (−9.68; 7.09)	0.064
Fat mass (g)	22	−0.31 (−1.81; 1.20)	0.672	18	−0.50 (−2.42; 1.41)	0.577	22	−7.21 (−25.53; 11.12)	0.417	22	8.08 (−321.65; 337.81)	0.959	18	7.63 (−1.77; 17.03)	0.100	**22**	**79.04 (0.22; 157.86)**	**0.049**
Lean mass (g)	22	−0.06 (−3.15; 3.04)	0.971	18	−0.49 (−3.94; 2.95)	0.761	22	−14.35 (−52.0; 23.30)	0.431	22	149.36 (−532.36; 831.08)	0.649	18	15.88 (0.80; 32.55)	0.060	**22**	**211.89 (63.92;359.87)**	**0.008**
**5 years**																		
Weight (g)	34	−0.77 (−5.37; 3.84)	0.736	28	1.84 (−2.06; 5.74)	0.339	32	−39.73 (−95.70; 16.23)	0.156	34	−153.73 (−1047.55;740.09)	0.727	**28**	**27.94 (0.98; 54.90)**	**0.043**	**32**	**257.23 (6.30;508.16)**	**0.045**
Fat mass (g)	23	0.18 (−1.83; 2.06)	0.901	20	−0.40 (−2.86; 2.06)	0.732	23	−10.41 (−36.21; 15.39)	0.407	23	32.90 (−324.78; 390.58)	0.848	20	7.98 (−5.53; 20.48)	0.226	23	51.72 (−57.67;161.11)	0.332
Lean mass (g)	23	−0.02 (−4.32; 4.29)	0.993	20	0.31 (−4.27; 4.88)	0.888	23	−34.76 (−90.29; 20.76)	0.204	23	−160.14 (−974.70; 654.40)	0.683	**20**	**28.56 (6.58; 50.54)**	**0.015**	23	181.40 (−58.58;421.38)	0.129

Adjusted linear regression analyses (Model 2): complete data set of associations is shown. Amino acid levels (µmol/g protein)-values for placental tissue (PL) were used; amino acid levels (µmol/L)-values for umbilical cord plasma (UC) and maternal plasma levels at week-32 of gestation (P32) were used. Beta (95% CI) values with *P*-values < 0.05 are given in bold. Abbreviations: BW, birthweight; N, sample numbers analyzed; *P*, *P*-value; PW, placental weight.

Model 2: adjusted for respective baseline levels of maternal blood plasma Tau and Trp at week-15 of gestation (P15) before treatment, sex, and group. Adjusted additionally for breastfeeding status (fully, partially breastfeeding/formula) from 1 year of age
