# Supplementary material for: Fetal sex modulates placental microRNA expression, potential microRNA-mRNA interactions, and levels of amino acid transporter expression and substrates: INFAT study subpopulation analysis of n-3 LCPUFA intervention during pregnancy and associations with offspring body composition
Source: BMC Mol Cell Biol. 2021 Mar 3;22:15. doi: 10.1186/s12860-021-00345-x (PMC7931339; doi:10.1186/s12860-021-00345-x)
Supplement: Supplementary file 5 — Additional file 5: Table S9. Oligonucleotides for RT-qPCR. [file 12860_2021_345_MOESM5_ESM.pdf]

Additional file 5

Table S9 Oligonucleotides for qPCR

**Gene name**		**Sequence (5´-3´)** Forward (F) / Reverse (R)	**Accession number**	**Primer location in exon**	**Product length (bp)**	**T (°C)**
hACTB	F	CCTGGAGAAGAGCTACGAGCTG	NM_001101.3	3-4	108	60
	R	GACTCCATGCCCAGGAAGGAAGG				
hPolR2a	F	CTTGTGTGATACCATGACCTGTCGTG	NM_000937.4	25-26	115	60
	R	GCACGTCCACCGTTTCCTCAAAGG				
hB2M	F	GGACTGGTCTTTCTATCTCTTGTAC	NM_004048.2	2-3	120	60
	R	TCTCGATCCCACTTAACTATCTTGG				
hTOP1	F	CTTCATCGACAAGCTTGCTCTG	NM_003286.2	14-15	98	60
	R	TGATGTGCTCCACACGAAGTGA				
SLC7A5	F	CCGTGAACTGCTACAGCGTGA	NM_003486.5	2-3	121	60
	R	ACATCACCCTTCCCGATCTGGA				
SLC6A6	F	TGGAGGTGCGTTTCTCATACCG	NM_001134367.1	3-5	157	60
	R	GGAGGCATAGCCGATACCAGA				
**Gene name**		**Order number (commercial primer)[†]**	**Accession number**	**Primer location in exon**	**Product length (bp)**	**T (°C)**
mTOR		QT00056133	NM_004958	51-52	66	55
**MicroRNA**		**MicroRNA sequence**	**Accession number**	**Assay ID[§]**	**Length (bp)**	**T (°C)**
RNU6b		CGCAAGGATGACACGCAAATTCGTGAAG CGTTCCATATTTTT	NR_002752	001093	n.s.	60
RNU24		ATTTGCTATCTGAGAGATGGTGATGACAT TTTAAACCACCAAGATCGCTGATGCA	NR_002447	001001	n.s.	60
hsa-mirR-26b		UUCAAGUAAUUCAGGAUAGGU	MI0000084	000407	n.s.	60
hsa-miR-99a		AACCCGUAGAUCCGAUCUUGUG	MI0000101	000435	n.s.	60
hsa-miR-100		AACCCGUAGAUCCGAACUUGUG	MI0000102	000437	n.s.	60
hsa-miR-375		UUUGUUCGUUCGGCUCGCGUGA	MI0000783	000564	n.s.	60
hsa-miR-30d		UGUAAACAUCCCCGACUGGAAG	MI0000255	000420	n.s.	60

†, commercial available primer ordered from Qiagen (Hilden, Germany); §, commercial primer ordered from Applied Biosystems (Darmstadt, Germany); n.s. = was not stated.
